# Syntheses and Structures of Functionalized Macrocyclic Carbon Nanohoops Bearing a [9]Cycloparaphenylene or a Higher Homological Unit

**DOI:** 10.1021/acsomega.4c11201

**Published:** 2025-01-16

**Authors:** Liu Li, Stephen M. Long, Brian S. Dolinar, Brian V. Popp, Kung K. Wang

**Affiliations:** C. Eugene Bennett Department of Chemistry, West Virginia University, Morgantown, West Virginia 26506, United States

## Abstract

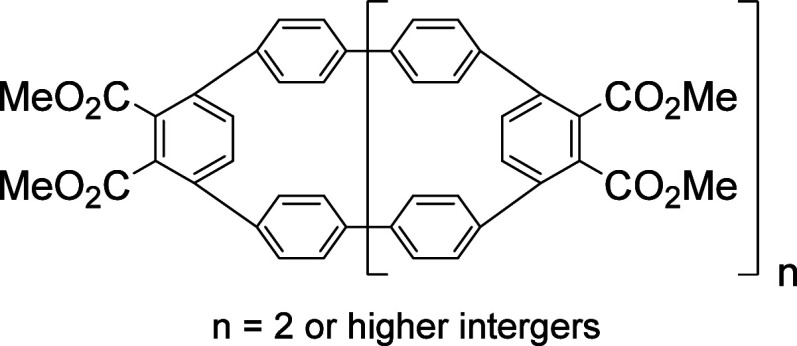

The Diels–Alder reactions between (*E*,*E*)-1,4-bis(4-bromophenyl)-1,3-butadiene and dienophiles and the use of the resulting adducts for macrocyclization and aromatization were established. Several functionalized macrocyclic structures bearing a functionalized [9]cycloparaphenylene or a higher homological unit were isolated. The distribution of the macrocyclic structures was found to be governed by the structures of the starting Diels–Alder adducts. An empirical correlation between the angles of the two 4-bromophenyl groups of the X-ray structures of the Diels–Alder adducts and the macrocyclization steps was observed.

## Introduction

**Scheme 1:**
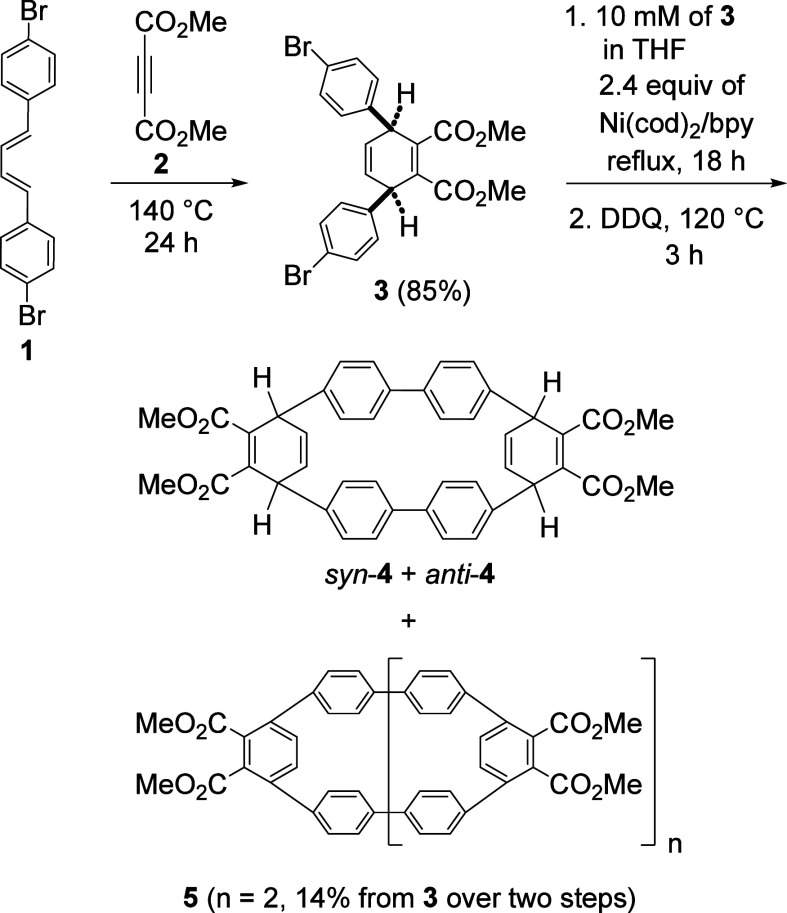
Synthesis of the Cyclic Dimers *syn*- and *anti*-**4** and a Functionalized Trimer [9]CPP **5**.

We earlier reported the Diels–Alder reaction between (*E*,*E*)-1,4-bis(4-bromophenyl)-1,3-butadiene (**1**) and dimethyl acetylenedicarboxylate (**2**) to produce the Diels–Alder adduct **3** (Scheme 1).^1^ The use of **3** for the Ni(cod)_2_ and 2,2′-bipyridyl (bpy)-mediated macrocyclization reaction and the subsequent 2,3-dichloro-5,6-dicyano-1,4-benzoquinone (DDQ)-promoted aromatization reaction were also investigated. The cyclic dimers *syn*- and *anti*-**4** were found to be the minor products, whereas a functionalized cyclic trimer **5** bearing a [9]cycloparaphenylene ([9]CPP) unit was found to be the major product. The use of Ni(cod)_2_/bpy for macrocyclization was reported previously.^2^ We have further investigated the effects of the amount of Ni(cod)_2_/bpy and the structures of the Diels–Alder adduct, derived from **1** and several different dienophiles, on the macrocyclization step and the influence of the DDQ-promoted aromatization reaction. The different structures of the Diels–Alder adduct could provide insights into the factor affecting the macrocyclization step.

## Results and Discussion

From the proposed reaction mechanisms of the Ni(cod)_2_/bpy-medicated homocoupling reaction,^3^ it could be expected that the macrocyclization step would be sensitive to the amount of Ni(cod)_2_/bpy used. We obtained Ni(cod)_2_ from two commercial sources, and various amounts of Ni(cod)_2_/bpy were used to promote macrocyclization. The use of 1.0, 1.5, 2.0, and 2.4 equiv of Ni(cod)_2_/bpy for macrocyclization was investigated. It was found that only trace amounts of dimers *syn*- and *anti*-**4** and trimer **5** were observed with 1.0 equiv of Ni(cod)_2_/bpy. By using 2.4 equiv of Ni(cod)_2_/bpy, the amount of trimer **5** (10% isolated) was diminished. However, with either 1.5 or 2.0 equiv of Ni(cod)_2_/bpy, trimer **5** was isolated in slightly enhanced yields. The amount of 1.5 equiv of Ni(cod)_2_/bpy appeared to produce a good result for macrocyclization, and therefore, this amount was used for further investigations.

As observed previously,^1^ small amounts (<1%) of the cyclic dimers *syn*- and *anti*-**4** were observed before oxidation with 2,3-dichloro-5,6-dicycno-1,4-benzoquinone (DDQ). Upon oxidation with 1.34 equiv of DDQ at 120 °C for 3 h, the ^1^H NMR spectrum of the crude reaction mixture showed the formation of trimer **5** (*n* = 2), tetramer **6** (*n* = 3), and pentamer **7** (*n* = 4) with singlets in the aromatic region at δ 6.88, 7.00, and 7.15 ppm in CDCl_3_ at room temperature, respectively, in a ratio of 100:26:19. However, the crude ^1^H NMR spectrum also showed incomplete aromatization with signals around δ 4.5 ppm of the diallylic hydrogens and δ 5.9 ppm of the alkenyl hydrogens. With an additional DDQ (2.23 equiv) and a longer reaction time of 18 h in refluxing chlorobenzene (bp = 132 °C), the aromatization step was complete with the absence of signals around δ 4.5 and 5.9 ppm (Supporting Information, Figure S1). In addition, the singlets of the crude ^1^H NMR spectrum in the methoxy region showing signals belonging to **5** to **8** and perhaps higher homologues were also observed. Trimer **5** (15%), tetramer **6** (4.7%), pentamer **7** (3.0%), and hexamer **8** (*n* = 5, 2.4%) were isolated for complete structural elucidation. It was observed previously that the ^1^H NMR spectrum of monomer **9**^1^ (*n* = 0 with hydrogen atoms at the ends) exhibited an aromatic singlet at δ 7.52 ppm and a methoxy singlet at δ 3.61 ppm. The empirical correlations suggest that as the ring becomes larger and the conformation of the macrocyclic structure becomes more like that of monomer **9**, the aromatic singlet becomes more downfield and the methoxy singlet becomes more upfield as anticipated.

**Scheme 2:**
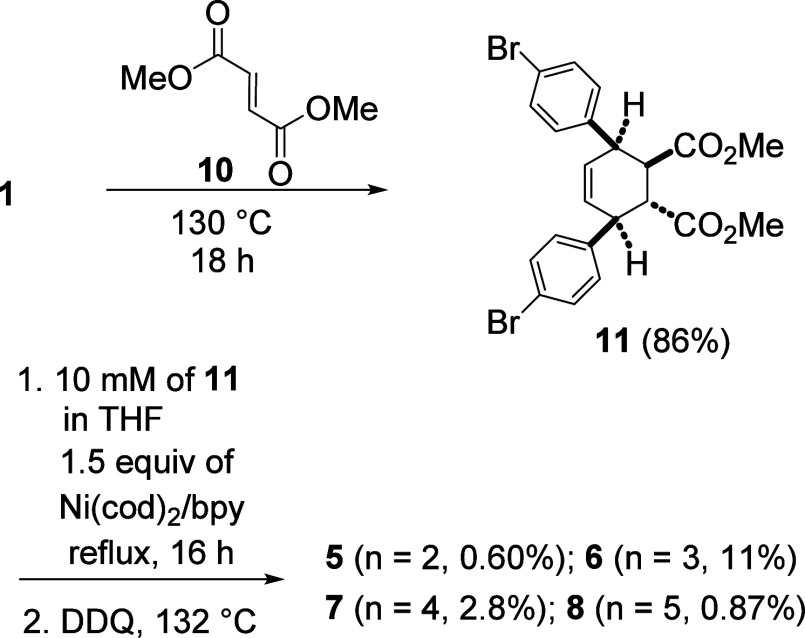
Macrocyclization and Aromatization of **11** to Form Functionalized Trimer [9]CPP **5**, Tetramer [12]CPP **6**, Pentamer [15]CPP **7**, and Hexamer [18]CPP **8**.

We have also investigated the Diels–Alder reaction between **1** and dimethyl fumarate (**10**)^4^ to form **11**(5) and the use of **11** for macrocyclization (Scheme 2). As in the case of **3**, the Diels–Alder reaction between **1** and **10** produced only one adduct **11**. The macrocylization reaction of **11** was mediated by 1.5 equiv of Ni(cod)_2_/bpy followed by the DDQ-promoted aromatization reaction. The ^1^H NMR spectrum of the crude reaction mixture clearly showed three singlets in the aromatic region belonging to trimer **5**, tetramer **6**, and pentamer **7** with the integration ratio of 11:100:36, respectively (Supporting Information, Figure S2). Similarly, the ^1^H NMR spectrum in the methoxy region showed singlets belonging to **5** to **8**. In addition, singlets that are shifted more upfield, presumably due to macrocycles of higher homologues, were also observed. We were able to isolate **5** (0.60%), **6** (11%), **7** (2.8%), and **8** (0.87%) for complete structural elucidation. It is interesting to note that, unlike **3**, tetramer **6** was obtained as the major homologue from **11**.

**Scheme 3:**
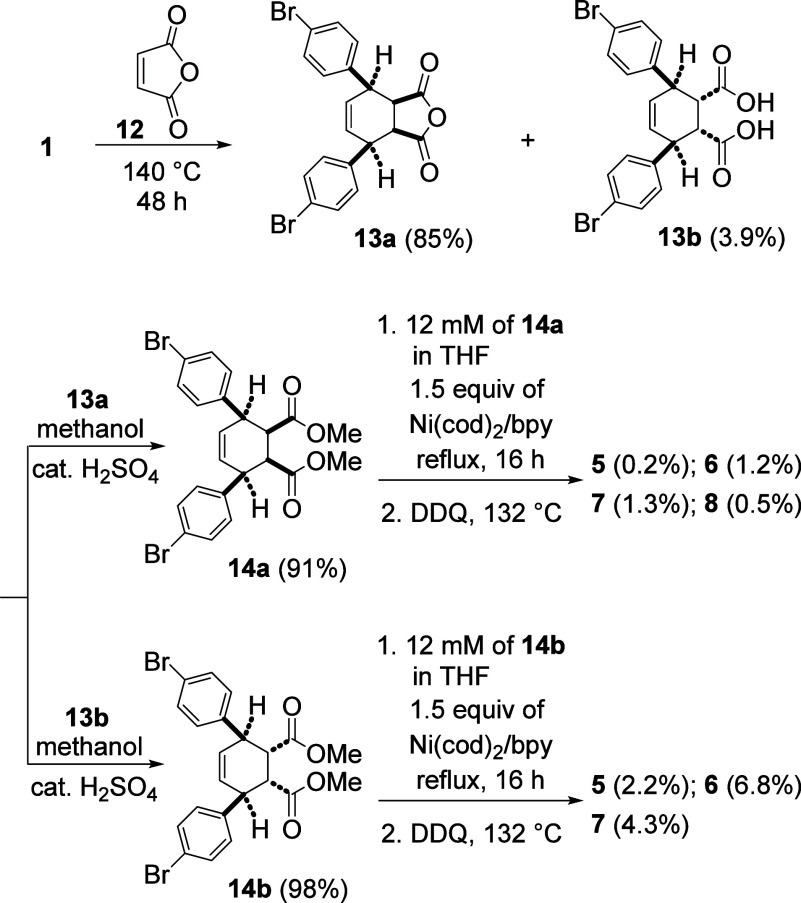
Macrocyclization and Aromatization of **14a** or **14b** to Form Trimer [9]CPP **5** to Hexamer [18]CPP **8**.

The Diels–Alder reaction between **1** and dimethyl maleate^4a,6^ gave no cycloaddition adduct at 130 °C for 18 h. However, the Diels–Alder reaction between **1** and maleic anhydride (**12**)^4,7^ was found to produce 85% of the *endo* adduct **13a** (Scheme 3). After silica gel column chromatography, diacid **13b** (3.9%) from the corresponding *exo* adduct was obtained. On treatment of **13a** and **13b** with methanol in the presence of a catalytic amount of concentrated sulfuric acid,^8^ the corresponding dimethyl ester **14a**(9) and **14b**(9) was obtained, respectively. When **14a** was treated with 1.5 equiv of Ni(cod)_2_/bpy followed by the DDQ-promoted aromatization reaction, the progress of the reaction was followed by the ^1^H NMR spectrum (Supporting Information, Figure S3). We were able to isolate trimer **5** to hexamer **8** in lower yields. With **14b**, the progress of the reaction was again monitored by the ^1^H NMR spectrum (Supporting Information, Figure S4), and higher yields of **5** to **7** were produced.

**Scheme 4:**
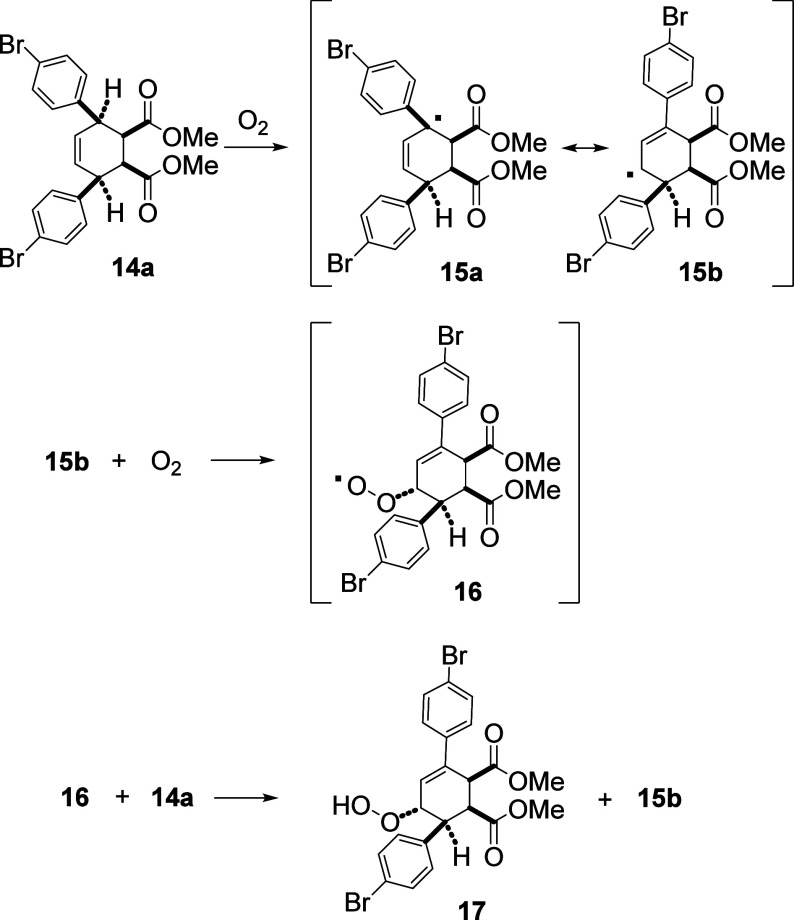
Autoxidation to Form Hydroperoxide **17**.

Dimethyl ester **14a** as a white solid was stable under air. However, when **14a** was dissolved in trichloromethane, it was found to be slowly oxidized by O_2_ to form hydroperoxide **17**(9) (Scheme 4) at room temperature. A free-radical mechanism, as shown in Scheme 4, was most likely responsible for the autoxidation.^10^ An initial reaction of **14a** with a triplet diatomic oxygen produced **15a**, which can also be regarded as **15b** for its resonance structure. The allylic hydrogens in **14a** are also benzylic, making the initiation step of the hydrogen-atom abstraction more feasible. Subsequent propagation steps involved a reaction with diatomic oxygen from the less hindered side of **15b** to produce **16** followed by hydrogen-atom abstraction from **14a** to produce **17** and free-radical **15b** again to restart the propagation steps. Because singlet diatomic oxygen was not generated, it is less likely that an ene reaction was involved.^11^ Interestingly, it was observed that **11** and **14b** were stable toward oxygen in a trichloromethane solution at room temperature. An explanation for such an observation perhaps could be found in the preferential conformations of the central cyclohexenyl rings of **11**, **14a**, and **14b**.^12^ The allylic hydrogens in **14a** could adopt preferentially the pseudoaxial orientations parallel to the C–C π bond, which is required for resonance stabilization. On the other hand, the allylic hydrogens in **11** and **14b** prefer pseudoequatorial positions in order to minimize steric interactions with the carbomethoxy groups. Such a conformation effect on the stability toward oxygen was proposed previously for the substituted 5-methylene-1,3-cyclohexadiene system.^13^ Nevertheless, the effect of the carbomethoxy groups on the stabilities of the transition states leading to radical species, such as those of **15a**/**15b**, could not be ruled out.

The bond angle of the two bonds attaching the two 4-bromophenyl groups to the central ring of **3** was reported to be 73.5° in the crystal lattice,^1^ whereas those of **11**,^5^**14a**,^9^ and **14b**(9) are 96.6°, 121.8°, and 79.0°, respectively. While the use of the smaller scale of **14b** may cause lower isolated yields of **5**, **6**, and **7** and the inability to isolate **8**, this observation appears to be in rough empirical correlation between the total yields of the macrocyclization step and the angles of the bonds connecting the two 4-bromophenyl groups to the central rings in the crystal lattice structures. The substantially larger angle of **14a** gave low yields of the macrocyclization step, as the formation of the rings became more unlikely.

## Conclusions

In summary, a synthetic pathway leading to macrocycles containing a functionalized [9]CPP, [12]CPP, [15]CPP, [18]CPP or perhaps higher homologues was established. The Diels–Alder reactions between (*E*,*E*)-1,4-bis(4-bromophenyl)-1,3-butadiene and dienophiles allowed the placing of the two 4-bromophenyl groups *cis* to each other in the six-membered rings, which was essential to the success of the subsequent Ni(cod)_2_/bpy-promoted macrocyclization step.^14^ The distribution of the macrocyclic structures from **5** to **8** and perhaps higher homologues was found to be governed by the structure of the starting Diels–Alder adduct. The bond angle of the two bonds attaching the two 4-bromophenyl groups to the central ring **3** in the X-ray structure appears to show an empirical correlation with the yields of the macrocyclization step.

## Experimental Section

### General Information

All reactions were conducted in oven-dried (120 °C) glassware under a nitrogen atmosphere. An oil bath was used as the heat source for the reactions that required heating. Chemicals, including Ni(cod)_2_, 2,2′-bipyridyl, 2,3-dichloro-5,6-dicyano-1,4-benzoquinone, dimethyl fumarate (**11**), and maleic anhydride (**12**), were purchased from chemical suppliers and were used as received. The reported procedure was used to prepare **3**.^1^ Chemical shifts of NMR spectra were reported as parts per million (δ) relative to the signal of CHCl_3_ at 7.26 ppm or DMSO-*d*_5_ at 2.50 ppm for ^1^H NMR spectra and the center line signal of the CDCl_3_ triplet at 77.0 ppm or DMSO-*d*_6_ at 39.5 ppm for ^13^C{^1^H} NMR spectra. Infrared (IR) spectra of solid samples were recorded on a Fourier transform IR system equipped with a diamond crystal attenuated total reflectance sampling interface. HRMS spectra were obtained on an Orbitrap mass analyzer coupled with electrospray ionization (ESI).

#### Experimental Procedure for **5**, **6**, **7**, and **8** from Dibromide **3**

To a 200 mL flask were added dibromide **3** (500 mg, 0.988 mmol) and 2,2′-bipyridyl (231 mg, 1.48 mmol). The flask was flushed with nitrogen and placed in a glovebox under a nitrogen atmosphere before Ni(cod)_2_ (408 mg, 1.48 mmol) and anhydrous THF (100 mL) were added. The flask was fitted with a condenser and a rubber septum and removed from the glovebox. The reaction mixture was heated at reflux for 16 h before it was allowed to cool to rt. Then, the reaction mixture was passed through a short pad of a silica gel column (4 cm) and eluted with a mixture of ethyl acetate and dichloromethane (1:1). The combined eluates were concentrated and dried *in vacuo*. The residue was treated with DDQ (500 mg, 2.20 mmol) in 10 mL of chlorobenzene at reflux for 18 h. Dichloromethane (50 mL) was added, and the reaction mixture was passed through a short, basic aluminum oxide column. The column was eluted with an additional 300 mL of a mixture of dichloromethane and ethyl acetate (1:1). The combined eluates were concentrated *in vacuo* (Figure S1), and the residue was purified by flash column chromatography (silica gel/dichloromethane:ethyl acetate = 96:4 to 70:30) to produce [9]CPP **5** (51 mg, 0.049 mmol, 15%, *R*_f_ = 0.5, dichloromethane/ethyl acetate = 95:5),^1^ [12]CPP **6** (16 mg, 0.011 mmol, 4.7%, *R*_f_ = 0.3, dichloromethane/ethyl acetate = 95:5), [15]CPP **7** (10 mg, 0.0058 mmol, 3.0%, *R*_f_ = 0.5, dichloromethane/ethyl acetate = 90:10), and [18]CPP **8** (8.2 mg, 0.0040 mmol, 2.4%, *R*_f_ = 0.4 with tailing likely due to poor solubility, dichloromethane/ethyl acetate = 90:10 and later more ethyl acetate) as white solids. **6**: IR 1727, 1232, 819 cm^–1^; ^1^H NMR (CDCl_3_, 400 MHz) δ 7.63 (d, *J* = 8.3 Hz, 16 H), 7.48 (d, *J* = 8.2 Hz, 16 H), 6.99 (s, 8 H), 3.83 (s, 24 H); ^13^C{^1^H} NMR (CDCl_3_, 100 MHz) δ 168.3, 139.8, 139.2, 139.0, 134.6, 130.0, 129.7, 126.9, 52.7; HRMS (ESI) *m*/*z* [M]^+^ calcd for C_88_H_64_O_16_ 1376.4189; found 1376.4243. **7**: ^1^H NMR (CDCl_3_, 400 MHz) δ 7.65 (d, *J* = 8.1 Hz, 20 H), 7.49 (d, *J* = 8.0 Hz, 20 H), 7.15 (s, 10 H), 3.81 (s, 30 H); ^13^C{^1^H} NMR (CDCl_3_, 100 MHz) δ 168.3, 139.9, 139.4, 139.1, 134.1, 130.4, 129.6, 127.0, 52.7; HRMS (ESI) *m*/*z* [M]^+^ calcd for C_110_H_80_O_20_ 1720.5238; found 1720.5267. **8**: ^1^H NMR (CDCl_3_, 400 MHz) δ 7.66 (d, *J* = 8.0 Hz, 24 H), 7.49 (d, *J* = 7.9 Hz, 24 H), 7.25 (s, 12 H), 3.80 (s, 36 H); ^13^C{^1^H} NMR (CDCl_3_, 100 MHz) δ 168.4, 139.9, 139.7, 139.1, 133.7, 130.7, 129.4, 127.1, 52.7; HRMS (ESI) *m*/*z* [M]^+^ calcd for C_132_H_96_O_24_ 2064.6286; found 2064.6272.

#### Experimental Procedure for Dibromide **11**

To a mixture of dimethyl fumarate (**10**, 790 mg, 5.49 mmol) and (*E*,*E*)-1,4-bis(4-bromophenyl)-1,3-butadiene (**1**, 1.00 g, 2.75 mmol) in a pressure glass tube was added 3 mL of dry toluene under a nitrogen atmosphere. The reaction mixture was stirred at 130 °C for 18 h before it was cooled to rt. Solvents were evaporated *in vacuo*, and the residue was subjected to flash column chromatography (silica gel/ethyl acetate:hexanes = 1:19) to provide **11** (1.20 g, 2.36 mmol, 86%) as a colorless oil: IR 1728, 1268, 808 cm^–1^; ^1^H NMR (CDCl_3_, 400 MHz) δ 7.47 (dd, *J* = 11.4, 8.2 Hz, 4 H), 7.10 (d, *J* = 8.3 Hz, 4 H), 5.89 (s, 2 H), 4.02 (dd, *J* = 5.8, 3.0 Hz, 1 H), 3.57 (d, *J* = 11.1 Hz, 1 H), 3.48 (dd, *J* = 11.7, 6.4 Hz, 1 H) 3.50 (s, 3 H), 3.40 (s, 3 H), 2.80 (t, *J* = 11.5 Hz, 1 H); ^13^C{^1^H} NMR (CDCl_3_, 100 MHz) δ 175.0, 172.1, 140.9, 137.9, 131.8, 131.6, 130.73, 130.70, 129.4, 128.0, 121.8, 121.2, 51.61, 51.57, 47.8, 46.2, 44.7, 41.9; HRMS (ESI) *m*/*z* [M + Na]^+^ calcd for C_22_H_20_Br_2_O_4_Na 528.9621, 530.9600, 532.9580; found 528.9623, 530.9601, 532.9579.

Recrystallization of **11** from a mixture of dichloromethane and hexanes produced a single crystal suitable for an X-ray structure analysis.

#### Experimental Procedure for **5**, **6**, **7**, and **8** from Dibromide **11**

To a 500 mL flask were added dibromide **11** (1.00 g, 1.97 mmol) and 2,2′-bipyridyl (461 mg, 2.95 mmol). The flask was flushed with nitrogen and placed in a glovebox under a nitrogen atmosphere before Ni(cod)_2_ (811 mg, 2.95 mmol) and anhydrous THF (200 mL) were added. The flask was fitted with a condenser and a rubber septum and then removed from the glovebox. The reaction mixture was heated at reflux for 16 h before it was allowed to cool to rt. Then, the reaction mixture was passed through a short pad of silica gel column (4 cm) and eluted with a mixture of ethyl acetate and dichloromethane (1:1). The combined eluates were concentrated and dried *in vacuo*. The residue was treated with DDQ (500 mg, 2.20 mmol) in 20 mL of chlorobenzene at reflux for 18 h. The progress of the reaction was monitored by a ^1^H NMR spectrum. An additional DDQ (1.00 g, 4.41 mmol) was added to the reaction mixture, which was allowed to run to completion. Dichloromethane (50 mL) was added, and the reaction mixture was passed through a short basic aluminum oxide column. The column was eluted with an additional 300 mL of a mixture of dichloromethane and ethyl acetate (1:1). The combined eluates were concentrated *in vacuo* (Figure S2), and the residue was purified by flash column chromatography (silica gel/dichloromethane:ethyl acetate = 97:3 to 70:30) to produce **5** (4.1 mg, 0.0040 mmol, 0.6%), **6** (75 mg, 0.054 mmol, 11%), **7** (19 mg, 0.011 mmol, 2.8%), and **8** (5.9 mg, 0.0029 mmol, 0.87%) as white solids.

#### Experimental Procedure for Dibromides **13a** and **13b**

To a mixture of maleic anhydride (**12**, 566 mg, 5.77 mmol) and (*E*,*E*)-1,4-bis(4-bromophenyl)-1,3-butadiene (**1**, 1.00 g, 2.75 mmol) in a pressure glass tube was added 8 mL of dry toluene under nitrogen. The reaction mixture was stirred at 140 °C for 48 h before it was cooled to rt. The white precipitate was collected by filtration and dried *in vacuo* to provide **13a** as a white solid (1.08 g, 2.34 mmol, 85%). The solvents from the filtrate were removed *in vacuo*, and the residue was subjected to flash column chromatography to provide **13b** (silica gel/ethyl acetate/hexanes = 15:85) as a white solid (51 mg, 0.106 mmol, 3.9%). **13a**: IR 1728, 1231, 819 cm^–1^; ^1^H NMR (DMSO-*d*_6_, 400 MHz) δ 7.58 (d, *J* = 8.6 Hz, 4 H), 7.36 (d, *J* = 8.0 Hz, 4 H), 6.53 (s, 2 H), 3.96 (m, 2 H), 3.89 (m, 2 H); ^13^C{^1^H} NMR (DMSO-*d*_6_, 100 MHz) δ 171.2, 138.4, 131.5, 131.1, 130.9, 120.1, 47.7, 39.8; HRMS (ESI) *m*/*z* [M + H]^+^ calcd for C_20_H_15_Br_2_O_3_ 460.9383, 462.9362, 464.9342; found 460.9384, 462.9367, 464.9342. **13b**: ^1^H NMR (CDCl_3_, 400 MHz) δ 7.49 (d, *J* = 8.0 Hz, 4 H), 7.21 (d, *J* = 8.1 Hz, 4 H), 5.84 (s, 2 H), 4.08 (d, *J* = 5.6 Hz, 2 H), 3.11 (d, *J* = 5.4 Hz, 2 H), 1.9–1.2 (s, br, 2 H); ^13^C{^1^H} NMR (DMSO-*d*_6_, 100 MHz) δ 173.1, 142.8, 131.5, 130.5, 128.8, 119.7, 45.2, 40.6; HRMS (ESI) *m*/*z* [M + Na]^+^ calcd for C_20_H_16_Br_2_O_4_Na 500.9308, 502.9287, 504.9267; found 500.9326, 502.9305, 504.9286.

#### Experimental Procedure for Dibromide **14a**

A solution of **13a** (400 mg, 0.866 mmol) in 10 mL of methanol and 0.3 mL of conc. H_2_SO_4_ was stirred at reflux for 4 h. Toluene (1.0 mL) was added, and the reflux was continued for an additional hour. The solvents were evaporated *in vacuo*, and the residue was partitioned between ethyl acetate and water. The organic layer was dried with Na_2_SO_4_ and evaporated *in vacuo* to afford **14a** (400 mg, 0.787 mmol, 91%) as colorless oil: IR 1729, 1268, 808 cm^–1^; ^1^H NMR (CDCl_3_, 400 MHz) δ 7.42 (d, *J* = 8.3 Hz, 4 H), 7.25 (d, *J* = 8.3 Hz, 4 H), 6.09 (s, 2 H), 3.92 (d, *J* = 7.1 Hz, 2 H), 3.45 (d, *J* = 7.3 Hz, 2 H), 3.31 (s, 6 H); ^13^C{^1^H} NMR (CDCl_3_, 100 MHz) δ 171.4, 139.3, 131.4, 130.9, 129.1, 121.0, 51.2, 45.6, 41.1; HRMS (ESI) *m*/*z* [M + H]^+^ calcd for C_22_H_21_Br_2_O_4_ 506.9801, 508.9781, 510.9760; found 506.9800, 508.9775, 510.9756.

Recrystallization of **14a** from a mixture of diethyl ether and hexanes under a nitrogen atmosphere produced a single crystal suitable for an X-ray structure analysis.

#### Experimental Procedure for **5**, **6**, **7**, and **8** from Dibromide **14a**

To a 200 mL flask were added dibromide **14a** (242 mg, 0.476 mmol) and 2,2′-bipyridyl (114 mg, 0.730 mmol). The flask was flushed with nitrogen and placed in a glovebox under a nitrogen atmosphere before Ni(cod)_2_ (196 mg, 0.714 mmol) and anhydrous THF (40 mL) were added. The flask was fitted with a condenser and a rubber septum and then removed from the glovebox. The reaction mixture was heated at reflux for 16 h before it was allowed to cool to rt. Then, the reaction mixture was passed through a short pad of silica gel column (4 cm) and eluted with a mixture of ethyl acetate and dichloromethane (1:1). The combined eluates were concentrated and dried *in vacuo*. The residue was treated with DDQ (250 mg, 1.10 mmol) in 10 mL of chlorobenzene at reflux for 18 h. Dichloromethane (50 mL) was added, and the reaction mixture was passed through a short, basic aluminum oxide column. The column was eluted with an additional 100 mL of a mixture of dichloromethane and ethyl acetate (1:1). The combined eluates were concentrated *in vacuo* (Figure S3), and the residue was purified by flash column chromatography (silica gel/dichloromethane/ethyl acetate = 97:3 to 70:30) to produce **5** (0.4 mg, 0.0004 mmol, 0.2%), **6** (2.0 mg, 0.0014 mmol 1.2%), **7** (2.2 mg, 0.0013 mmol, 1.3%), and **8** (0.8 mg, 0.0004 mmol, 0.5%) as white solids.

#### Experimental Procedure for Dibromide **14b**

A solution of **13b** (50 mg, 0.104 mmol) in 2 mL of methanol and 0.1 mL of conc. H_2_SO_4_ was stirred at reflux for 4 h. Toluene (1.0 mL) was added, and the reflux was continued for an additional 1 h. The solvents were evaporated *in vacuo*, and the residue was partitioned between ethyl acetate and water. The organic layer was dried with Na_2_SO_4_ and evaporated *in vacuo* to afford **14b** (52 mg, 0.102 mmol, 98%) as colorless oil: ^1^H NMR (CDCl_3_, 400 MHz) δ 7.49 (d, *J* = 8.4 Hz, 4 H), 7.22 (d, *J* = 8.4 Hz, 4 H), 5.86 (s, 2 H), 4.01 (d, *J* = 5.2 Hz, 2 H), 3.63 (s, 6 H), 3.03 (d, *J* = 5.5 Hz, 2 H); ^13^C{^1^H} NMR (CDCl_3_, 100 MHz) δ 172.5, 141.8, 131.8, 130.1, 128.8, 120.9, 52.1, 45.8, 40.9; HRMS (ESI) *m*/*z* [M + H]^+^ calcd for C_22_H_21_Br_2_O_4_ 506.9801, 508.9781, 510.9760; found 506.9824, 508.9799, 510.9781.

Recrystallization of **14b** from a mixture of diethyl ether and hexanes produced a single crystal suitable for an X-ray structure analysis.

#### Experimental Procedure for **5**, **6**, and **7** from Dibromide **14b**

To a 50 mL flask were added dibromide **14b** (48 mg, 0.094 mmol) and 2,2′-bipyridyl (22 mg, 0.141 mmol). The flask was flushed with nitrogen and placed in a glovebox under a nitrogen atmosphere before Ni(cod)_2_ (39 mg, 0.142 mmol) and anhydrous THF (8 mL) were added. The flask was fitted with a condenser and a rubber septum and then removed from the glovebox. The reaction mixture was heated at reflux for 16 h before it was allowed to cool to rt. Then, the reaction mixture was passed through a short pad of silica gel column (4 cm) and eluted with a mixture of ethyl acetate and dichloromethane (1:1). The combined eluates were concentrated and dried *in vacuo*. The residue was treated with DDQ (100 mg, 0.441 mmol) in 3 mL of chlorobenzene at reflux for 18 h. An additional DDQ (100 mg, 0.441 mmol) was added to the reaction mixture, and the reaction was allowed to run for another 48 h. Dichloromethane (10 mL) was added, and the reaction mixture was passed through a short basic aluminum oxide column. The column was eluted with an additional 100 mL of a mixture of dichloromethane and ethyl acetate (1:1). The combined eluates were concentrated *in vacuo* (Figure S4), and the residue was purified by flash column chromatography (silica gel/dichloromethane/ethyl acetate = 97:3 to 85:15) to produce **5** (0.7 mg, 0.0007 mmol, 2.2%), **6** (2.2 mg, 0.0016 mmol, 6.8%), and **7** (1.4 mg, 0.0008 mmol, 4.3%) as white solids.

#### Experimental Procedure for Hydroperoxide **17** from Dibromide **14a**

To a 50 mL flask containing **14a** (200 mg, 0.394 mmol) was added 5 mL of acetone and 5 mL of chloroform. The solution was kept under air at rt for 20 days, and additional solvents were added to the flask to avoid the complete evaporation of solvents. The reaction mixture was concentrated *in vacuo*, and the residue was purified by flash column chromatography (silica gel/dichloromethane/ethyl acetate = 15:85) to produce hydroperoxide **17** (129 mg, 0.239 mmol, 61%) as a colorless oil: IR 3326 (br), 1740, 1709, 1202, 820 cm^–1^; ^1^H NMR (CDCl_3_, 400 MHz) δ 7.77 (s, 1H), 7.47 (d, *J* = 8.3 Hz, 2 H), 7.44 (d, *J* = 8.4 Hz, 2 H), 7.26 (d, *J* = 8 Hz, 2 H), 7.25 (d, *J* = 8 Hz, 2 H), 6.38 (t, *J* = 2.2 Hz, 1 H), 5.48 (dt, *J* = 7.5, 2.6 Hz, 1 H), 4.32 (d, *J* = 5.9 Hz, 1 H), 3.64 (dt, *J* = 7.4, 3.6 Hz, 1 H), 3.58 (s, 3 H), 3.52 (dd, *J* = 5.9, 3.8 Hz, 1 H), 3.28 (s, 3H); ^13^C{^1^H} NMR (CDCl_3_, 100 MHz) δ 171.3, 170.6, 139.2, 138.0, 137.4, 131.60, 131.57, 130.5, 127.3, 127.0, 121.9, 121.4, 81.3, 52.1, 51.9, 47.2, 46.4, 41.9; HRMS (ESI) *m*/*z* [M + Na]^+^ calcd for C_22_H_20_Br_2_O_6_Na 560.9519, 562.9498, 564.9478; found 560.9540, 562.9518, 564.9498.

Recrystallization of hydroperoxide **17** from a mixture of diethyl ether and hexanes produced a single crystal suitable for an X-ray structure analysis.

## Data Availability

The data underling this study are available in the published article and its online Supporting Information.

## Supporting Information Available

Single-crystal X-ray crystallographic structures; NMR spectra for new compounds (PDF)

## Accession Codes

CCDC 2383402–2383405 contain the supplementary crystallographic data for this paper. These data can be obtained free of charge via www.ccdc.cam.ac.uk/data_request/cif, or by emailing data_request@ccdc.cam.ac.uk, or by contacting The Cambridge Crystallographic Data Centre, 12 Union Road, Cambridge CB2 1EZ, UK; fax: + 44 1223 336033.
