# Supplementary material for: Syntheses and Structures of Functionalized Macrocyclic Carbon Nanohoops Bearing a [9]Cycloparaphenylene or a Higher Homological Unit
Source: ACS Omega. 2025 Jan 16;10(3):3222–7. doi: 10.1021/acsomega.4c11201 (PMC11780456; doi:10.1021/acsomega.4c11201)
Supplement: Supplementary file 1 — ao4c11201_si_001.pdf [file ao4c11201_si_001.pdf]

Syntheses and Structures of Functionalized Macrocyclic Carbon Nanohoops Bearing a [9]Cycloparaphenylene or a Higher Homological Unit

Liu Li, Stephen M. Long, Brian S. Dolinar, Brian V. Popp,

and Kung K. Wang*

C. Eugene Bennett Department of Chemistry, West Virginia University

Morgantown, West Virginia 26506, United States

Supporting Information

Table of Contents

Page

S3 Figure S1. The ^{1}H NMR Spectrum of the Crude Reaction Mixture from Dibromide **3** for **5**, **6**, **7**, **8**, and Possibly Higher Homologs.

S4 Figure S2. The ^{1}H NMR Spectrum of the Crude Reaction Mixture from Dibromide **11** for **5**, **6**, **7**, **8**, and Possibly Higher Homologs.

S5 Figure S3. The ^{1}H NMR Spectrum of the Crude Reaction Mixture from Dibromide **14a** for **5**, **6**, **7**, **8**, and Possibly Higher Homologs.

S6 Figure S4. The ^{1}H NMR Spectrum of the Crude Reaction Mixture from Dibromide **14b** for **5**, **6**, **7**, and Possibly Higher Homologs.

S7 Figure S5. Thermal Ellipsoid Plot of the Crystal Structure of Dibromide **11**.

S8−S9 Description of the X-ray Structural Analysis of Dibromide **11**.

S10 Figure S6. Thermal Ellipsoid Plot of the Crystal Structure of Dibromide **14a**.

S11−S12 Description of the X-ray Structural Analysis of Dibromide **14a**.

S13	Figure S7. Thermal Ellipsoid Plot of the Crystal Structure of Dibromide **14b**.
S14−S15	Description of the X-ray Structural Analysis of Dibromide **14b**.
S16	Figure S8. Thermal Ellipsoid Plot of the Crystal Structure of Hydroperoxide **17**.
S17−S18	Description of the X-ray Structural Analysis of Hydroperoxide **17**.
S19	References Cited
S20−S21	^{1}H and ^{13}C NMR Spectra of Macrocycle **6**.
S22−S23	^{1}H and ^{13}C NMR Spectra of Macrocycle **7**.
S24−S25	^{1}H and ^{13}C NMR Spectra of Macrocycle **8**.
S26−S27	^{1}H and ^{13}C NMR Spectra of Dibromide **11**.
S28−S29	^{1}H and ^{13}C NMR Spectra of Dibromide **13a**.
S30−S31	^{1}H and ^{13}C NMR Spectra of Dibromide **13b**.
S32−S33	^{1}H and ^{13}C NMR Spectra of Dibromide **14a**.
S34−S35	^{1}H and ^{13}C NMR Spectra of Dibromide **14b**.
S36−S37	^{1}H and ^{13}C NMR Spectra of Hydroperoxide **17**.

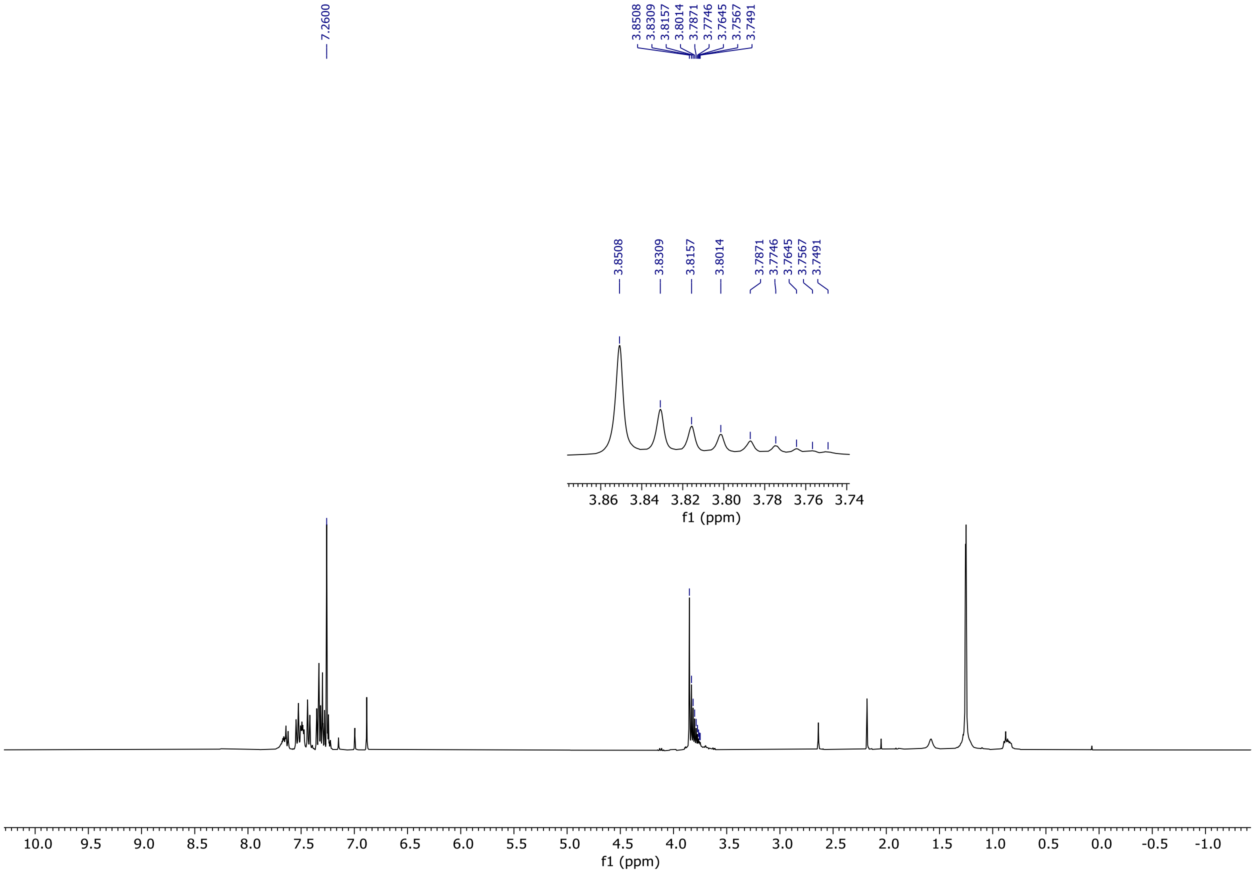

Figure S1. The ^{1}H NMR Spectrum of the Crude Reaction Mixture from Dibromide **3** for **5**, **6**, **7**, **8**, and Possibly Higher Homologs.

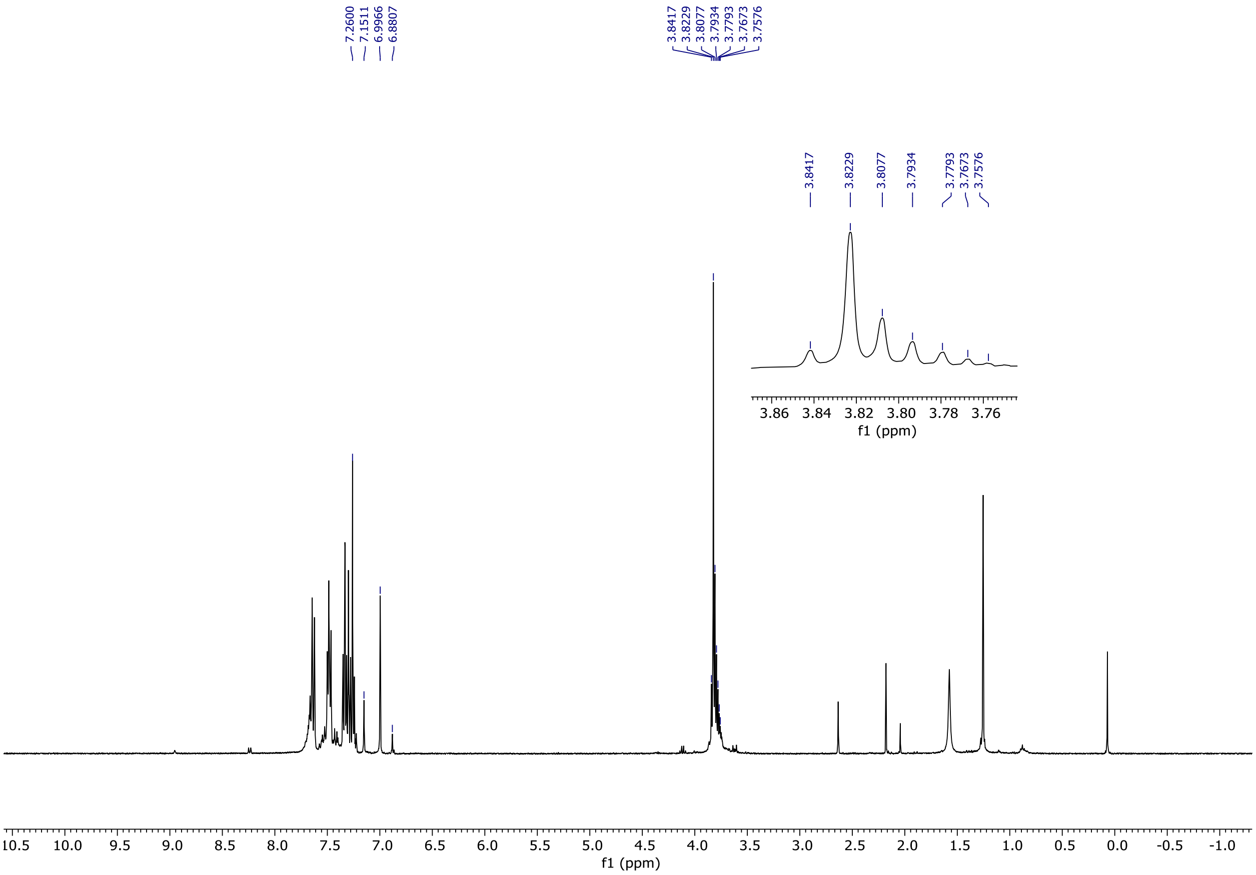

Figure S2. The ^{1}H NMR Spectrum of the Crude Reaction Mixture from Dibromide **11** for **5**, **6**, **7**, **8**, and Possibly Higher Homologs.

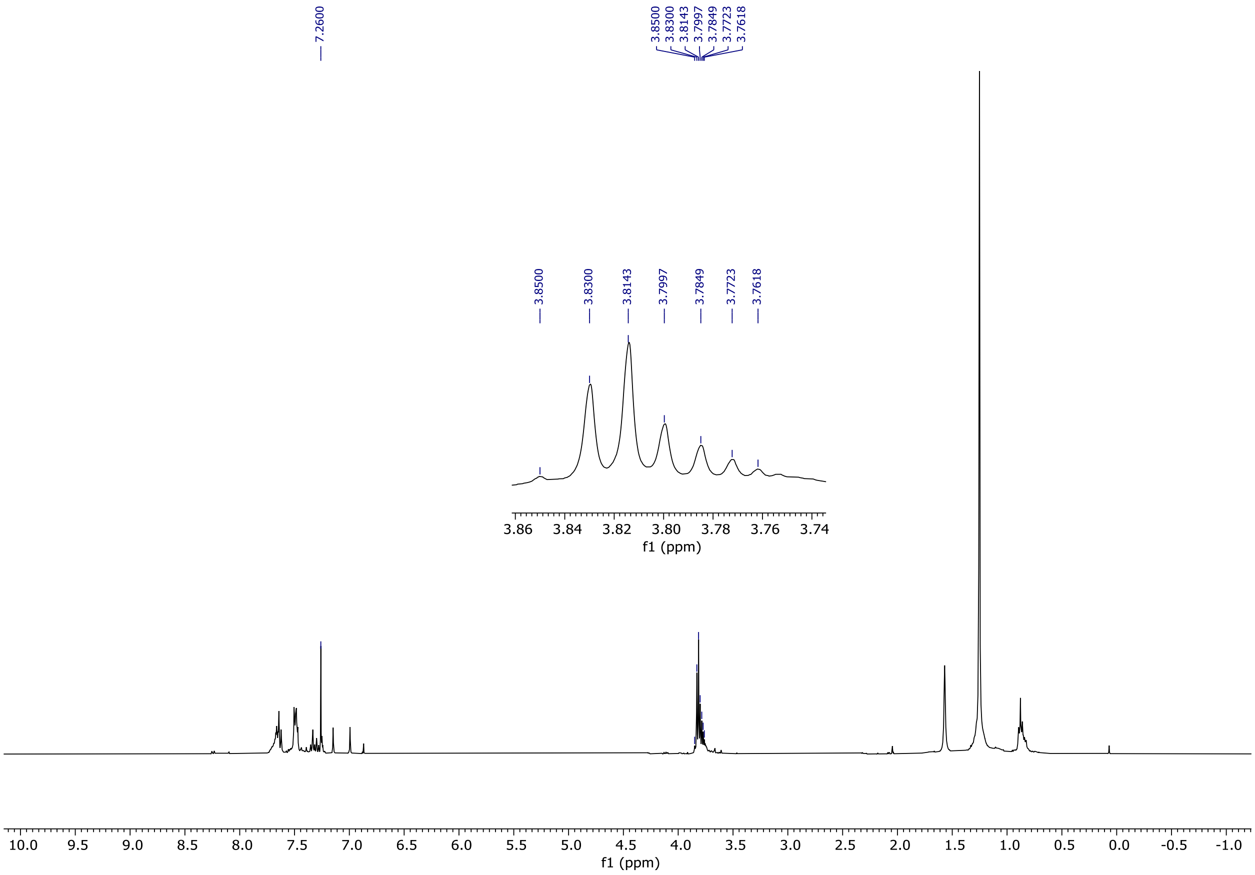

Figure S3. The ^{1}H NMR Spectrum of the Crude Reaction Mixture from Dibromide **14a** for **5**, **6**, **7**, **8**, and Possibly Higher Homologs.

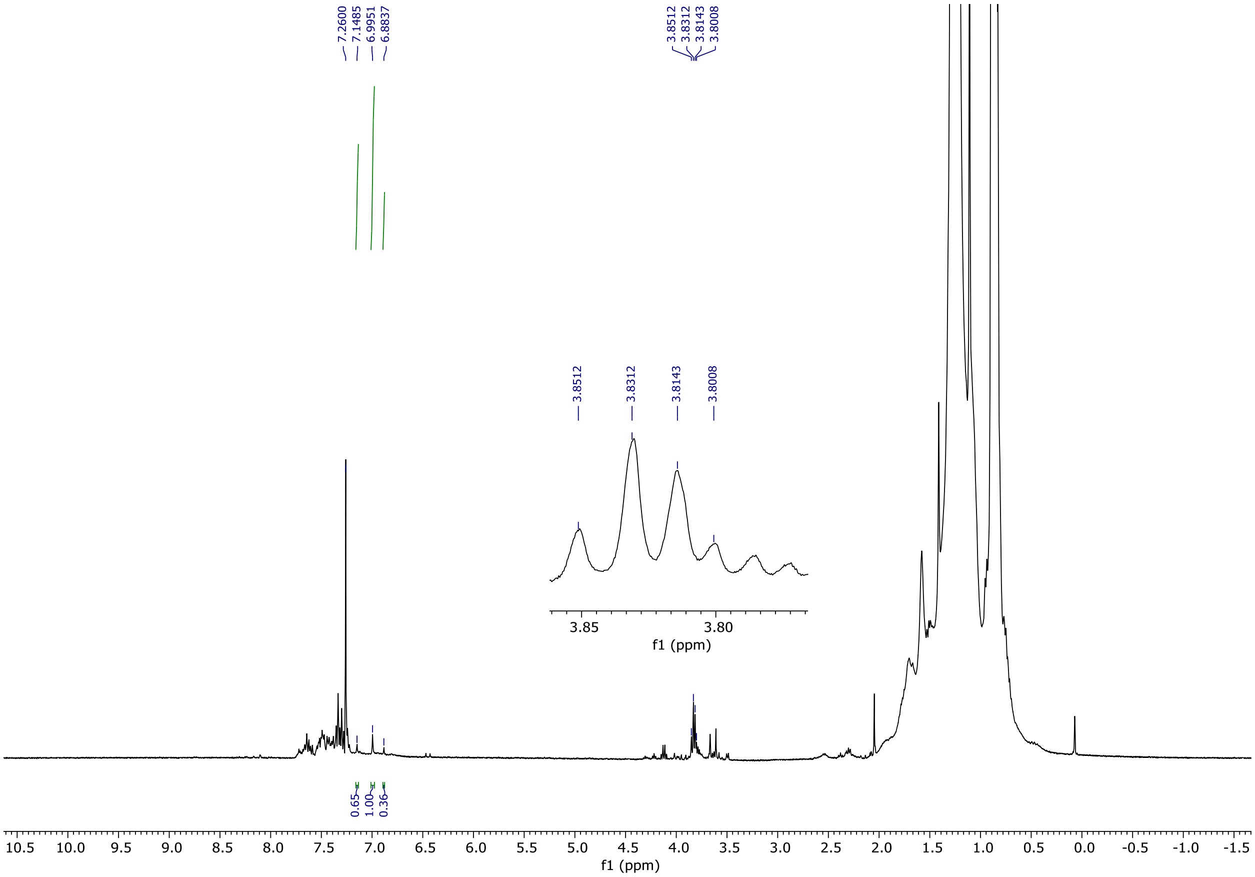

Figure S4. The ^{1}H NMR Spectrum of the Crude Reaction Mixture from Dibromide **14b** for **5**, **6**, **7**, and Possibly Higher Homologs.

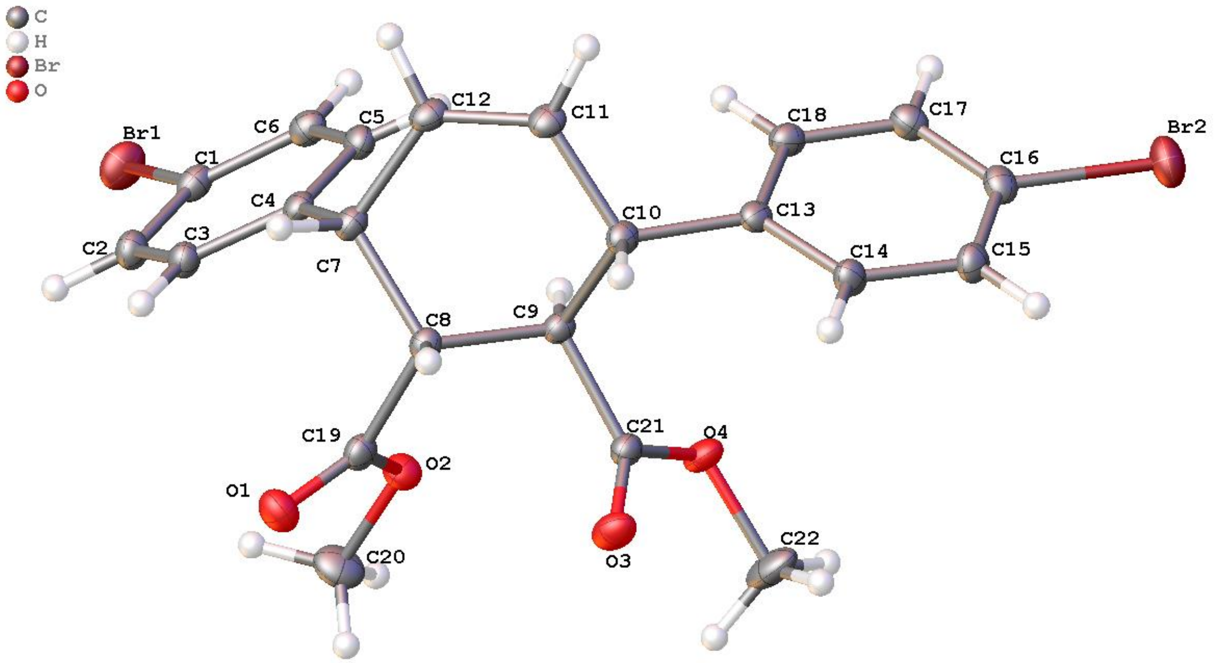

Figure S5. Thermal Ellipsoid Plot of the Crystal Structure of Dibromide **11**. All Non-hydrogen Atoms Are Drawn As 50% Thermal Probability Ellipsoids.

Description of the X-ray Structural Analysis of Dibromide **11**.

A colorless block-shaped crystal measuring 0.607 mm x 0.505 mm x 0.234 mm was selected under polybutene oil using a MiTeGen Micromount and mounted at 100(1) K to a D8-Venture diffractometer equipped with a Mo sealed tube X-ray source, a Triumph monochromator, and a Photon 2 CMOS area detector. The unit cell was determined from reflections harvested with a signal to noise ratio (I/σ) of at least 10 from a series of 2 ω scans of 6° with 0.5° frames using APEX3.[S1] The data were collected to a resolution of 0.6 Å using 7 ω and 1 φ scans.

The data were integrated using SAINT and corrected for absorption using SADABS.[S2] The systematic absences and E-statistics of the data were consistent with the space group $P2_1/n$. The structure was solved using the intrinsic phasing routine of SHELXT.[S3] The non-hydrogen atoms were located from a Fourier difference map of the electron density and anisotropically refined using the least-squares algorithm of SHELXL.[S4] The hydrogen atoms were then placed in calculated positions and refined with riding thermal parameters.

The final structure consisted of 255 parameters refined against 9973 independent reflections, giving refinement residuals of R_1 = 0.0396 (based off F^2 for $I > 2\sigma$) and wR_2 = 0.0758 (based of F^2 for all reflections). The final difference Fourier map was featureless.

Table 1. Crystal data and structure refinement for dibromide 11.

Identification code	kw45
Empirical formula	$C_{22}H_{20}Br_2O_4$
Formula weight	508.20
Temperature/K	100.0
Crystal system	Monoclinic
Space group	$P2_1/n$
a/Å	8.0864(2)
b/Å	10.9633(3)
c/Å	23.1821(6)
α/°	90
β/°	90.6130(10)
γ/°	90
Volume/Å^3	2055.06(9)
Z	4
ρ_{calc}g/cm^3	1.643
μ/mm^{-1}	3.970
F(000)	1016.0
Crystal size/mm^3	0.607 × 0.505 × 0.234
Radiation	Mo Kα (λ = 0.71073)
2Θ range for data collection/°	5.114 to 72.794
Index ranges	$-13 \leq h \leq 13$, $-18 \leq k \leq 18$, $-36 \leq l \leq 38$
Reflections collected	149651
Independent reflections	9973 [R_{int} = 0.0505, R_{sigma} = 0.0287]
Data/restraints/parameters	9973/0/255
Goodness-of-fit on F^2	1.117
Final R indexes [I>=2σ (I)]	R_1 = 0.0396, wR_2 = 0.0705
Final R indexes [all data]	R_1 = 0.0611, wR_2 = 0.0758
Largest diff. peak/hole / e Å^{-3}	0.65/-0.86

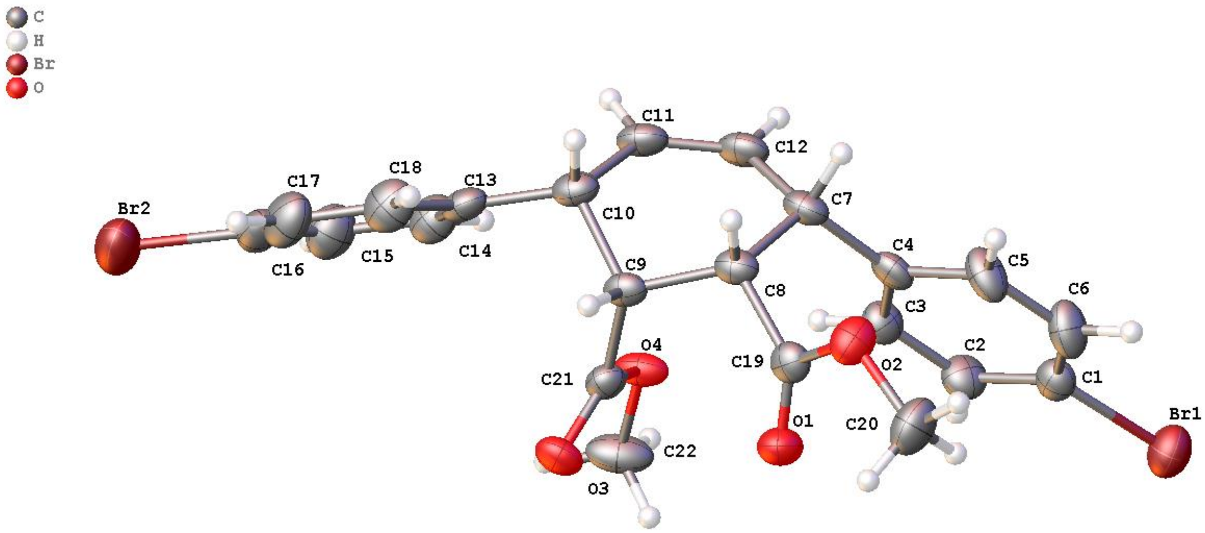

Figure S6. Thermal Ellipsoid Plot of the Crystal Structure of Dibromide **14a**. All Non-hydrogen Atoms Are Drawn As 50% Thermal Probability Ellipsoids.

Description of the X-ray Structural Analysis of Dibromide **14a**.

A colorless block-shaped crystal measuring 0.363 mm x 0.326 mm x 0.308 mm was selected under polybutene oil using a MiTeGen Micromount and mounted at 200(1) K to a D8-Venture diffractometer equipped with a Mo sealed tube X-ray source, a Triumph monochromator, and a Photon 2 CMOS area detector. The unit cell was determined from reflections harvested with a signal to noise ratio (I/σ) of at least 10 from a series of 2 ω scans of 6° with 0.5° frames using APEX3.[S1] A complete set of data was collected to a resolution of 0.75 Å using 6 ω and 1 φ scans.

The data were integrated using SAINT and corrected for absorption using SADABS.[S2] The systematic absences and E-statistics of the data were uniquely consistent with the space group $P2_1/c$. The structure was solved using the intrinsic phasing routine of SHELXT.[S3] The non-hydrogen atoms were located from a Fourier difference map of the electron density and anisotropically refined using the least-squares algorithm of SHELXL.[S4] The hydrogen atoms were then placed in calculated positions and refined with riding thermal parameters.

The final structure consisted of 255 parameters refined against 5128 independent reflections, giving refinement residuals of $R_1 = 0.0415$ (based off F^2 for $I > 2\sigma$) and $wR_2 = 0.0802$ (based of F^2 for all reflections). The final difference Fourier map was featureless.

Table 2. Crystal data and structure refinement for dibromide 14a.

Identification code	kw46_a
Empirical formula	$C_{22}H_{20}Br_2O_4$
Formula weight	508.20
Temperature/K	200.0
Crystal system	Monoclinic
Space group	$P2_1/c$
a/Å	24.3960(18)
b/Å	11.1057(9)
c/Å	7.4869(6)
α/°	90
β/°	94.411(5)
γ/°	90
Volume/Å^3	2022.5(3)
Z	4
ρ_{calc}g/cm^3	1.669
μ/mm^{-1}	4.034
F(000)	1016.0
Crystal size/mm^3	0.363 × 0.326 × 0.308
Radiation	Mo Kα (λ = 0.71073)
2Θ range for data collection/°	5.024 to 57.98
Index ranges	$-32 \leq h \leq 32$, $-15 \leq k \leq 14$, $-10 \leq l \leq 9$
Reflections collected	76064
Independent reflections	5128 [R_{int} = 0.0594, R_{sigma} = 0.0229]
Data/restraints/parameters	5128/0/255
Goodness-of-fit on F^2	1.148
Final R indexes [I>=2σ (I)]	R_1 = 0.0415, wR_2 = 0.0752
Final R indexes [all data]	R_1 = 0.0593, wR_2 = 0.0802
Largest diff. peak/hole / e Å^{-3}	0.58/-0.56

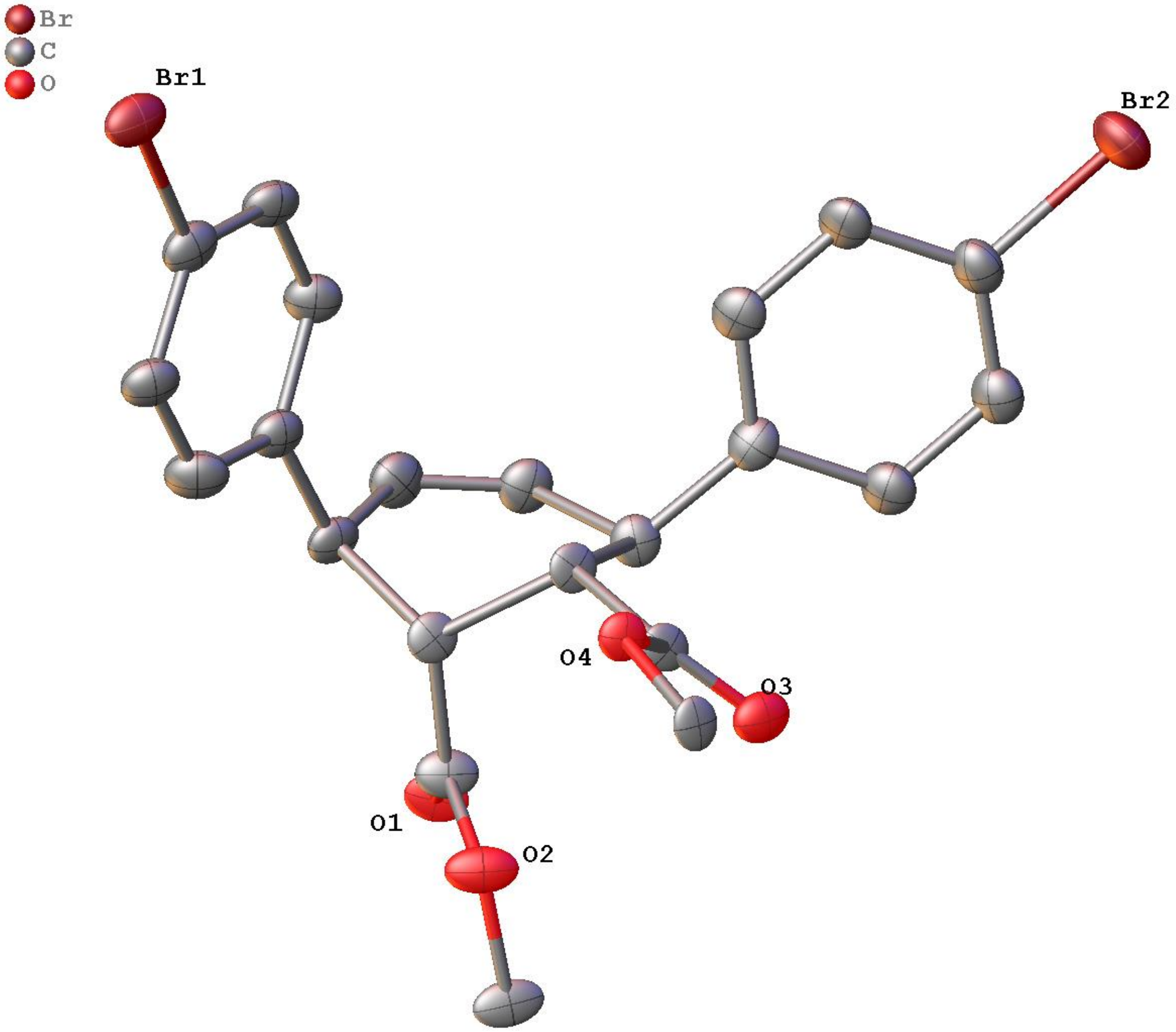

Figure S7. Thermal Ellipsoid Plot of the Crystal Structure of Dibromide **14b**. All Non-hydrogen Atoms Are Drawn As 50% Thermal Probability Ellipsoids. All Hydrogen Atoms Are Omitted for Clarity.

Description of the X-ray Structural Analysis of Dibromide **14b**.

A colorless needle-shaped crystal measuring 0.234 mm x 0.081 mm x 0.034 mm was selected under polybutene oil using a MiTeGen Micromount and mounted at 100(1) K to a D8-Venture diffractometer equipped with a Mo sealed tube X-ray source, a Triumph monochromator, and a Photon 2 CMOS area detector. The unit cell was determined from reflections harvested with a signal to noise ratio (I/σ) of at least 10 from a series of 2 ω scans of 6° with 0.5° frames using APEX3.[S1] A complete set of data was collected to a resolution of 0.82 Å using 4 ω scans.

The data were integrated using SAINT and corrected for absorption using SADABS.[S2] The systematic absences and E-statistics of the data were uniquely consistent with the space group $P2_1/n$. The structure was solved using the intrinsic phasing routine of SHELXT.[S3] The non-hydrogen atoms were located from a Fourier difference map of the electron density and anisotropically refined using the least-squares algorithm of SHELXL.[S4] The hydrogen atoms were then placed in calculated positions and refined with riding thermal parameters.

The structure exhibited whole molecule disorder (major component: 67.8(5)%). The geometries of the two positions of the molecule were restrained using the SAME command and the thermal parameters of all atoms were restrained using the SIMU command in SHELXL. In addition, several atoms on the carbonyl groups (C9 C9A O4 O4A C16A C16) were restrained using the ISOR command in order to ensure a chemically reasonable and computationally stable refinement.

The final structure consisted of 501 parameters refined against 3814 independent reflections, giving refinement residuals of $R_1 = 0.0744$ (based off F^2 for $I > 2\sigma$) and $wR_2 = 0.1599$ (based of F^2 for all reflections). The final difference Fourier map was featureless.

Table 3. Crystal data and structure refinement for dibromide 14b.

Identification code	kw47_0m_a
Empirical formula	$C_{22}H_{20}O_4Br_2$
Formula weight	508.20
Temperature/K	100.0
Crystal system	Monoclinic
Space group	$P2_1/n$
a/Å	7.7349(9)
b/Å	16.854(2)
c/Å	15.6306(19)
α/°	90
β/°	92.053(4)
γ/°	90
Volume/Å^3	2036.3(4)
Z	4
ρ_{calc}g/cm^3	1.658
μ/mm^{-1}	4.006
F(000)	1016.0
Crystal size/mm^3	0.234 × 0.081 × 0.034
Radiation	Mo Kα (λ = 0.71073)
2Θ range for data collection/°	4.834 to 51.328
Index ranges	-9 ≤ h ≤ 9, -20 ≤ k ≤ 20, -19 ≤ l ≤ 12
Reflections collected	8171
Independent reflections	3814 [R_{int} = 0.1114, R_{sigma} = 0.1760]
Data/restraints/parameters	3814/972/501
Goodness-of-fit on F^2	1.017
Final R indexes [I>=2σ (I)]	R_1 = 0.0744, wR_2 = 0.1343
Final R indexes [all data]	R_1 = 0.1685, wR_2 = 0.1599
Largest diff. peak/hole / e Å^{-3}	0.57/-0.63

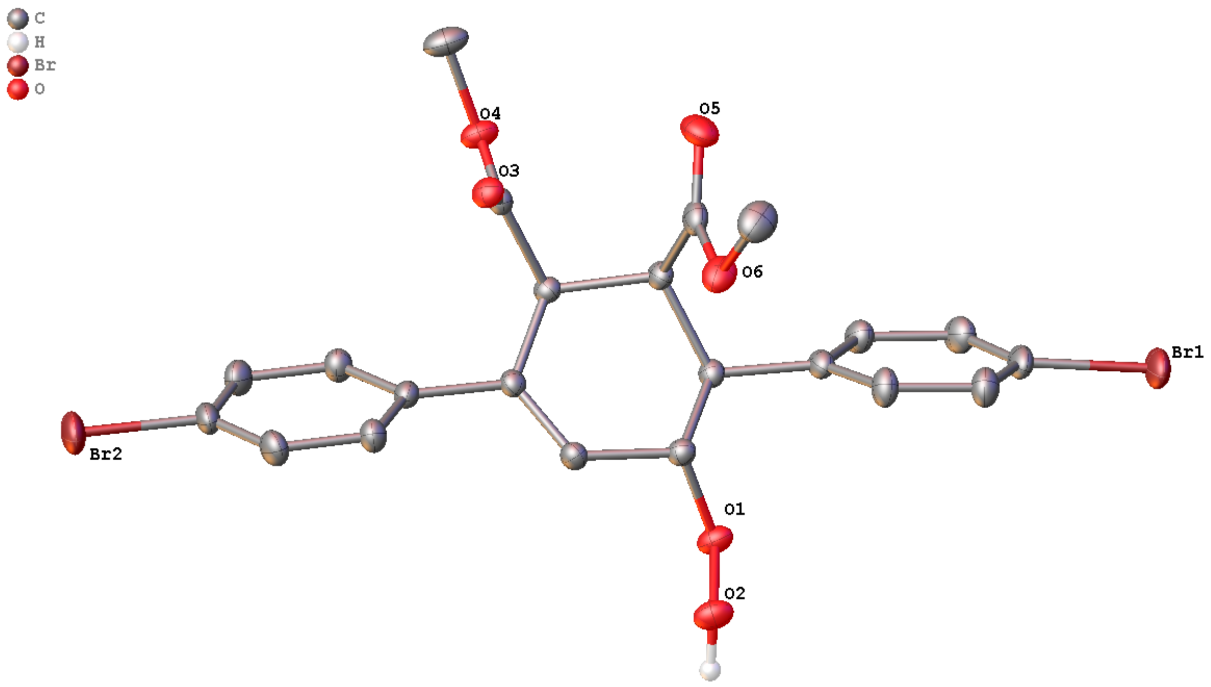

Figure S8. Thermal Ellipsoid Plot of the Crystal Structure of Hydroperoxide **17**. All Non-hydrogen Atoms Are Drawn As 50% Thermal Probability Ellipsoids. All Hydrogen Atoms, Except the One On the Oxygen Atom of the Hydroperoxide, Are Omitted for Clarity.

Description of the X-ray Structural Analysis of Hydroperoxide **17**.

A colorless block-shaped crystal measuring 0.406 mm x 0.352 mm x 0.200 mm was selected under polybutene oil using a MiTeGen Micromount and mounted at 100(1) K to a D8-Venture diffractometer equipped with a Mo sealed tube X-ray source, a Triumph monochromator, and a Photon 2 CMOS area detector. The unit cell was determined from reflections harvested with a signal to noise ratio (I/σ) of at least 10 from a series of 2 ω scans of 6° with 0.5° frames using APEX3.[S1] A complete set of data was collected to a resolution of 0.70 Å using 6 ω and 1 φ scans.

The data were integrated using SAINT and corrected for absorption using SADABS.[S2] The systematic absences and E-statistics of the data were uniquely consistent with the space group $P2_1/c$. The structure was solved using the intrinsic phasing routine of SHELXT.[S3] The non-hydrogen atoms were located from a Fourier difference map of the electron density and anisotropically refined using the least-squares algorithm of SHELXL.[S4] The hydrogen atoms were then placed in calculated positions and refined with riding thermal parameters.

Bromophenyl rings Br1 and Br2 both exhibited positional disorder (major occupancies: 94.7(4)% and 97.3(3)%, respectively). These were modeled with geometry and thermal parameter restraints in order to ensure a chemically reasonable and computationally stable refinement.

The final structure consisted of 384 parameters refined against 6550 independent reflections, giving refinement residuals of $R_1 = 0.0366$ (based off F^2 for $I > 2\sigma$) and $wR_2 = 0.0842$ (based of F^2 for all reflections). The final difference Fourier map was featureless.

Table 4. Crystal data and structure refinement for hydroperoxide 17.

Identification code	kw48_a
Empirical formula	$C_{22}H_{20}Br_2O_6$
Formula weight	540.20
Temperature/K	100.0
Crystal system	monoclinic
Space group	$P2_1/c$
a/Å	22.5207(5)
b/Å	6.12870(10)
c/Å	15.5150(3)
α/°	90
β/°	93.7630(10)
γ/°	90
Volume/$Å^3$	2136.80(7)
Z	4
$\rho_{calc}g/cm^3$	1.679
μ/mm^{-1}	3.830
F(000)	1080.0
Crystal size/mm^3	0.406 × 0.352 × 0.200
Radiation	Mo Kα (λ = 0.71073)
2Θ range for data collection/°	5.438 to 61.122
Index ranges	$-32 \leq h \leq 32$, $-8 \leq k \leq 8$, $-22 \leq l \leq 22$
Reflections collected	105572
Independent reflections	6550 [R_{int} = 0.0555, R_{sigma} = 0.0193]
Data/restraints/parameters	6550/378/384
Goodness-of-fit on F^2	1.072
Final R indexes [I>=2σ (I)]	R_1 = 0.0366, wR_2 = 0.0794
Final R indexes [all data]	R_1 = 0.0478, wR_2 = 0.0842
Largest diff. peak/hole / e $Å^{-3}$	0.89/-0.41

References Cited

S1 Bruker-AXS (2016). APEX 3 version 2016.9-0. Madison, Wisconsin, USA

S2. (a) Bruker-AXS (2015). *SAINT V8.37A.* Madison, Wisconsin, USA. (b) Bruker-AXS (2014). *SADABS*, Madison, Wisconsin, USA.

S3. Sheldrick, G. M. *Acta Crystallogr. A* **2015**, *A71*, 3-8.

S4. (a) Sheldrick, G. M. *Acta Crystallogr. C* **2015**, *C71*, 3-8.; (b) Dolomanov, O. V.; Bourhis, L. J.; Gildea, R. J.; Howard, J. A. K.; Puschmann, H. *J. Appl. Crystallogr.* **2009**, *42*, 339-341.

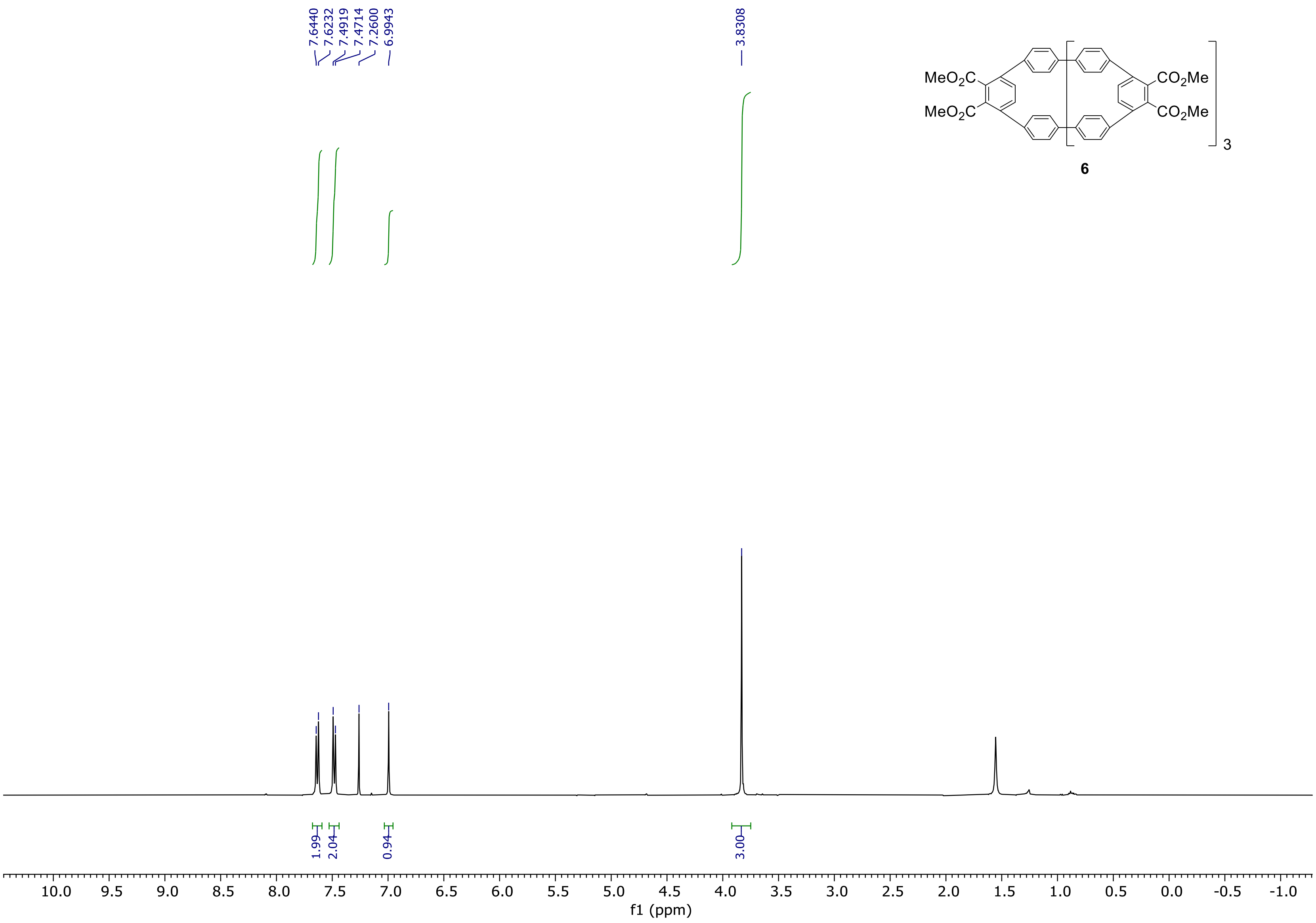
7.6440
7.6232
7.4919
7.4714
7.2600
6.9943
3.8308
MeO2C
MeO2C
CO2Me
CO2Me
3
6
1.99
2.04
0.94
3.00
10.0 9.5 9.0 8.5 8.0 7.5 7.0 6.5 6.0 5.5 5.0 4.5 4.0 3.5 3.0 2.5 2.0 1.5 1.0 0.5 0.0 -0.5 -1.0
f1 (ppm)

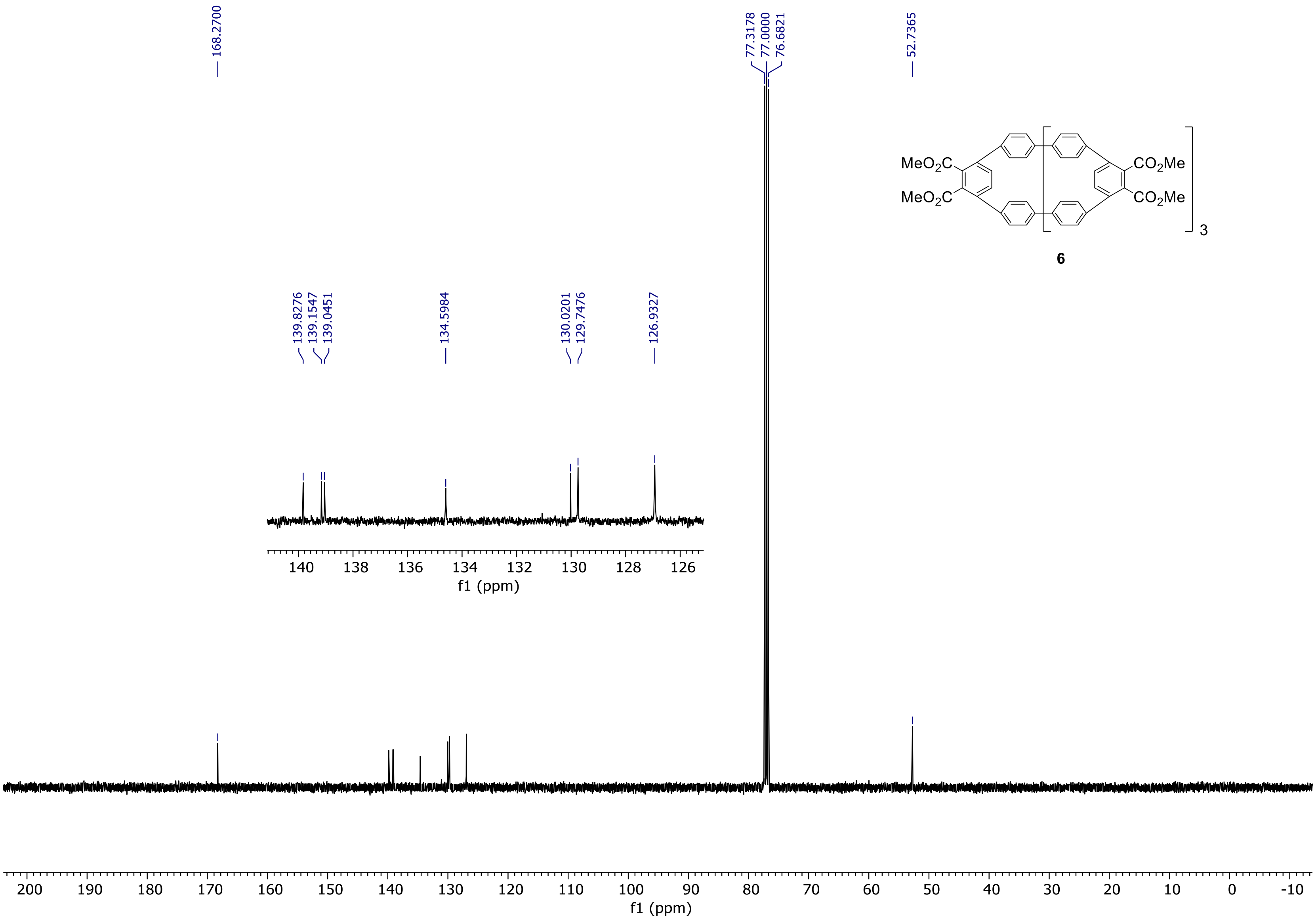

168.2700
77.3178
77.0000
76.6821
52.7365
139.8276
139.1547
139.0451
134.5984
130.0201
129.7476
126.9327
MeO2C
CO2Me
3
6
f1 (ppm)
f1 (ppm)

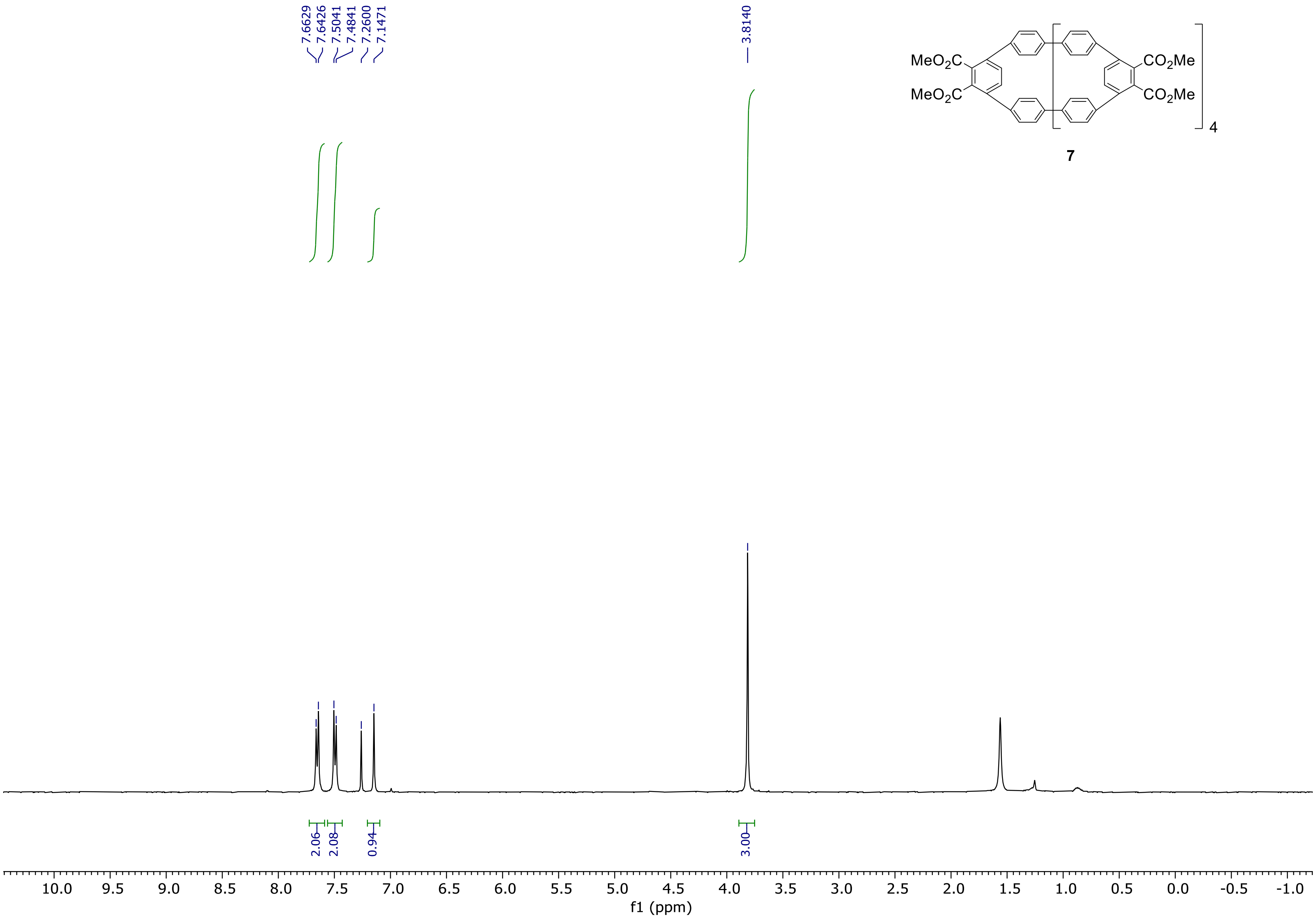
7.6629
7.6426
7.5041
7.4841
7.2600
7.1471
3.8140
MeO2C
MeO2C
CO2Me
CO2Me
4
2.06
2.08
0.94
3.00
10.0
9.5
9.0
8.5
8.0
7.5
7.0
6.5
6.0
5.5
5.0
4.5
4.0
3.5
3.0
2.5
2.0
1.5
1.0
0.5
0.0
-0.5
-1.0
f1 (ppm)

7

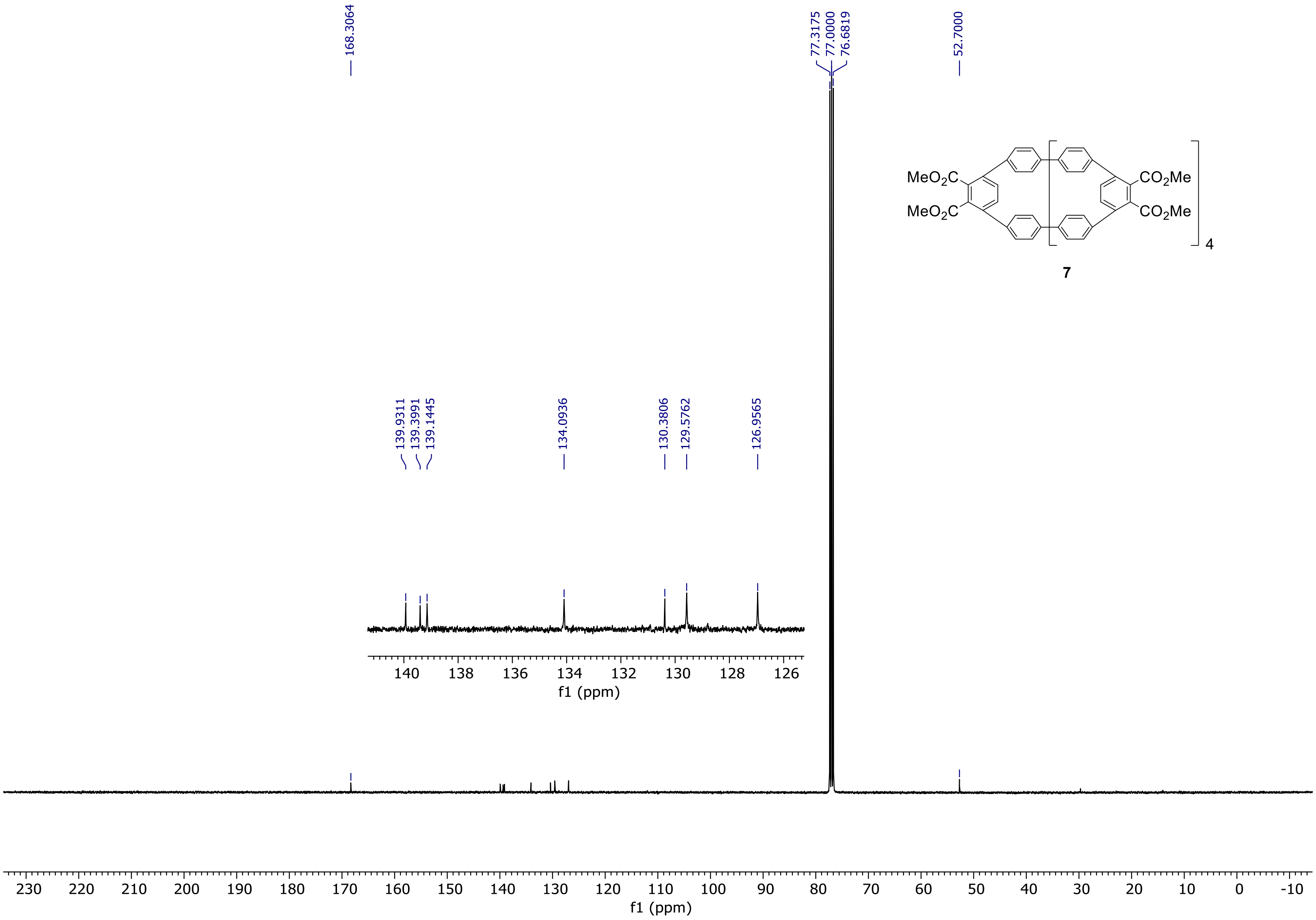

168.3064
77.3175
77.0000
76.6819
52.7000
139.9311
139.3991
139.1445
134.0936
130.3806
129.5762
126.9565
140 138 136 134 132 130 128 126
f1 (ppm)
230 220 210 200 190 180 170 160 150 140 130 120 110 100 90 80 70 60 50 40 30 20 10 0 -10
f1 (ppm)
MeO_2C
MeO_2C
CO_2Me
CO_2Me
4

7

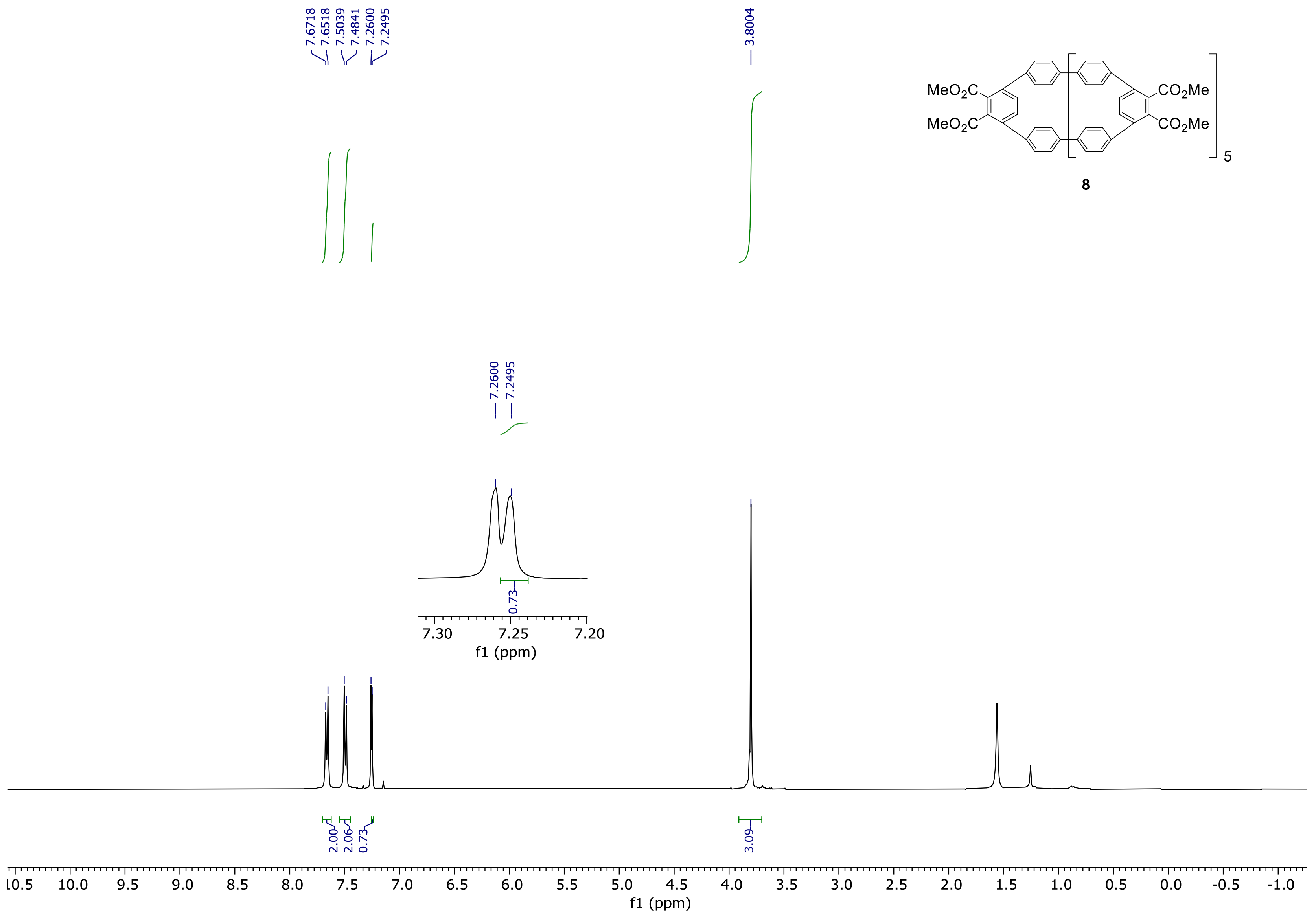
7.6718
7.6518
7.5039
7.4841
7.2600
7.2495
3.8004
MeO2C
MeO2C
CO2Me
CO2Me
5
8
7.2600
7.2495
0.73
7.30
7.25
7.20
f1 (ppm)
2.00
2.06
0.73
3.09
10.5 10.0 9.5 9.0 8.5 8.0 7.5 7.0 6.5 6.0 5.5 5.0 4.5 4.0 3.5 3.0 2.5 2.0 1.5 1.0 0.5 0.0 -0.5 -1.0
f1 (ppm)

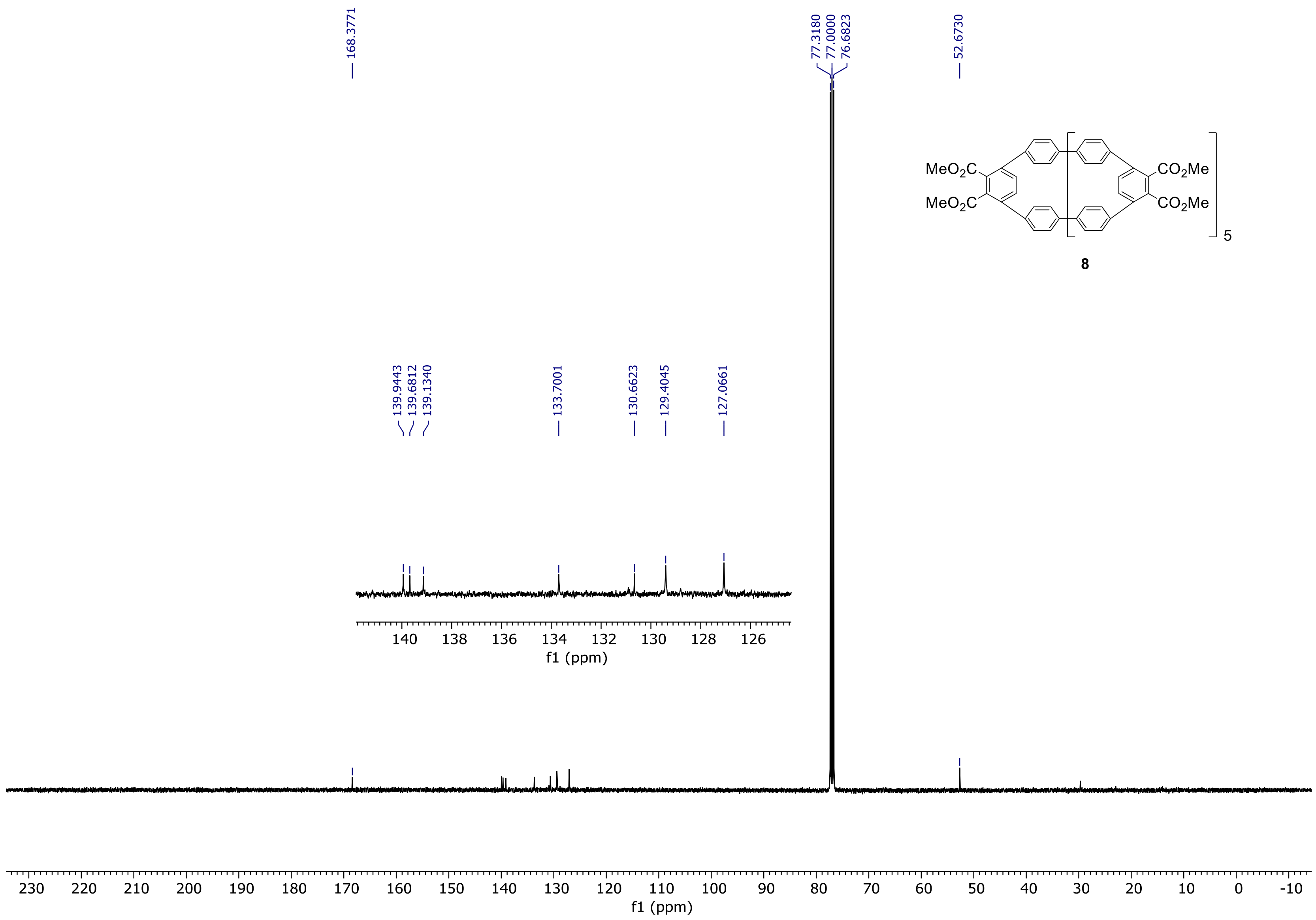
168.3771
77.3180
77.0000
76.6823
52.6730
139.9443
139.6812
139.1340
133.7001
130.6623
129.4045
127.0661
140 138 136 134 132 130 128 126
f1 (ppm)
230 220 210 200 190 180 170 160 150 140 130 120 110 100 90 80 70 60 50 40 30 20 10 0 -10
f1 (ppm)
MeO2C
MeO2C
CO2Me
CO2Me
5
8

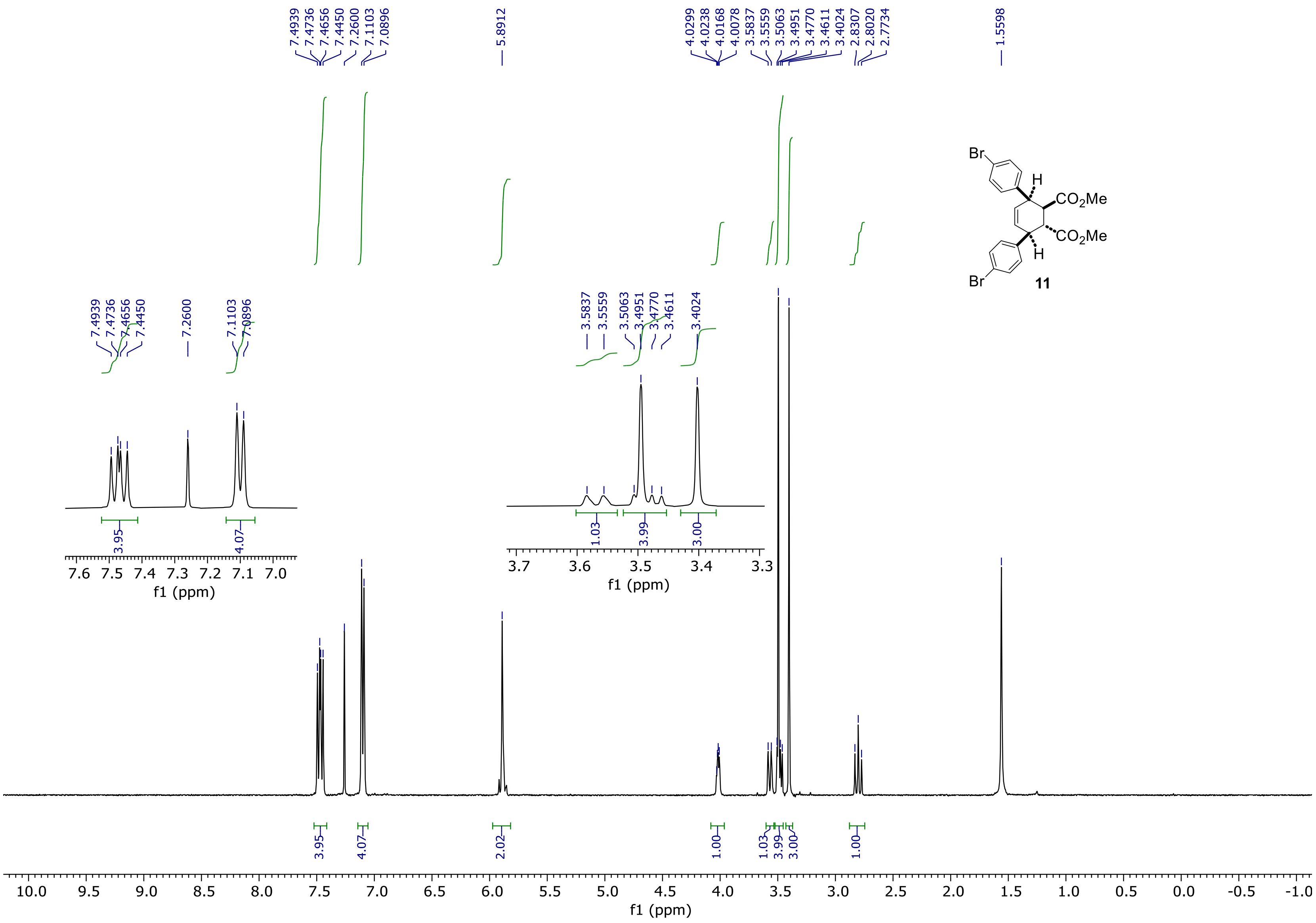

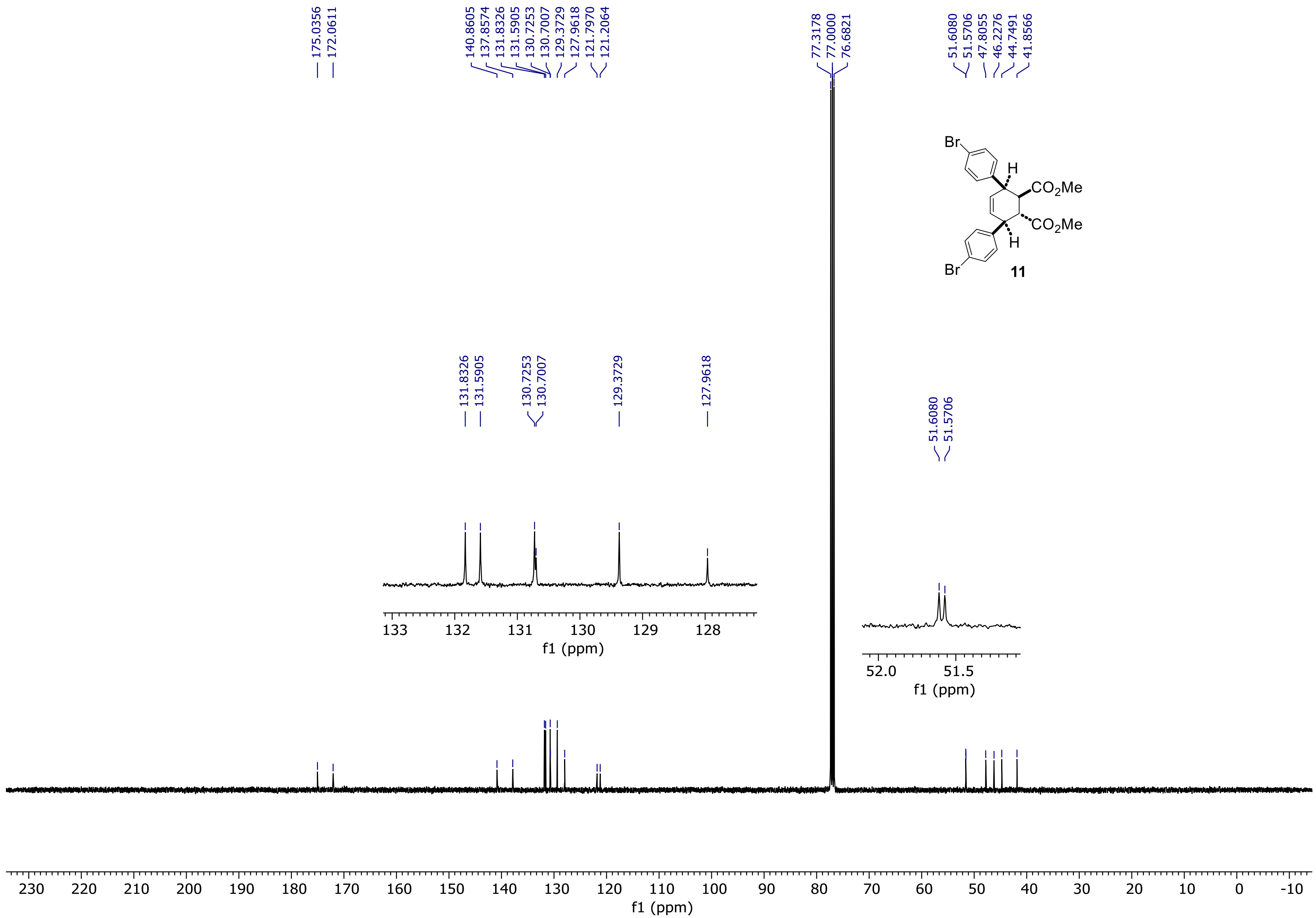
175.0356
172.0611
140.8605
137.8574
131.8326
131.5905
130.7253
130.7007
129.3729
127.9618
121.7970
121.2064
77.3178
77.0000
76.6821
51.6080
51.5706
47.8055
46.2276
44.7491
41.8566
Br
H
CO_2Me
CO_2Me
H
Br
11
131.8326
131.5905
130.7253
130.7007
129.3729
127.9618
133 132 131 130 129 128
f1 (ppm)
51.6080
51.5706
52.0 51.5
f1 (ppm)
230 220 210 200 190 180 170 160 150 140 130 120 110 100 90 80 70 60 50 40 30 20 10 0 -10
f1 (ppm)

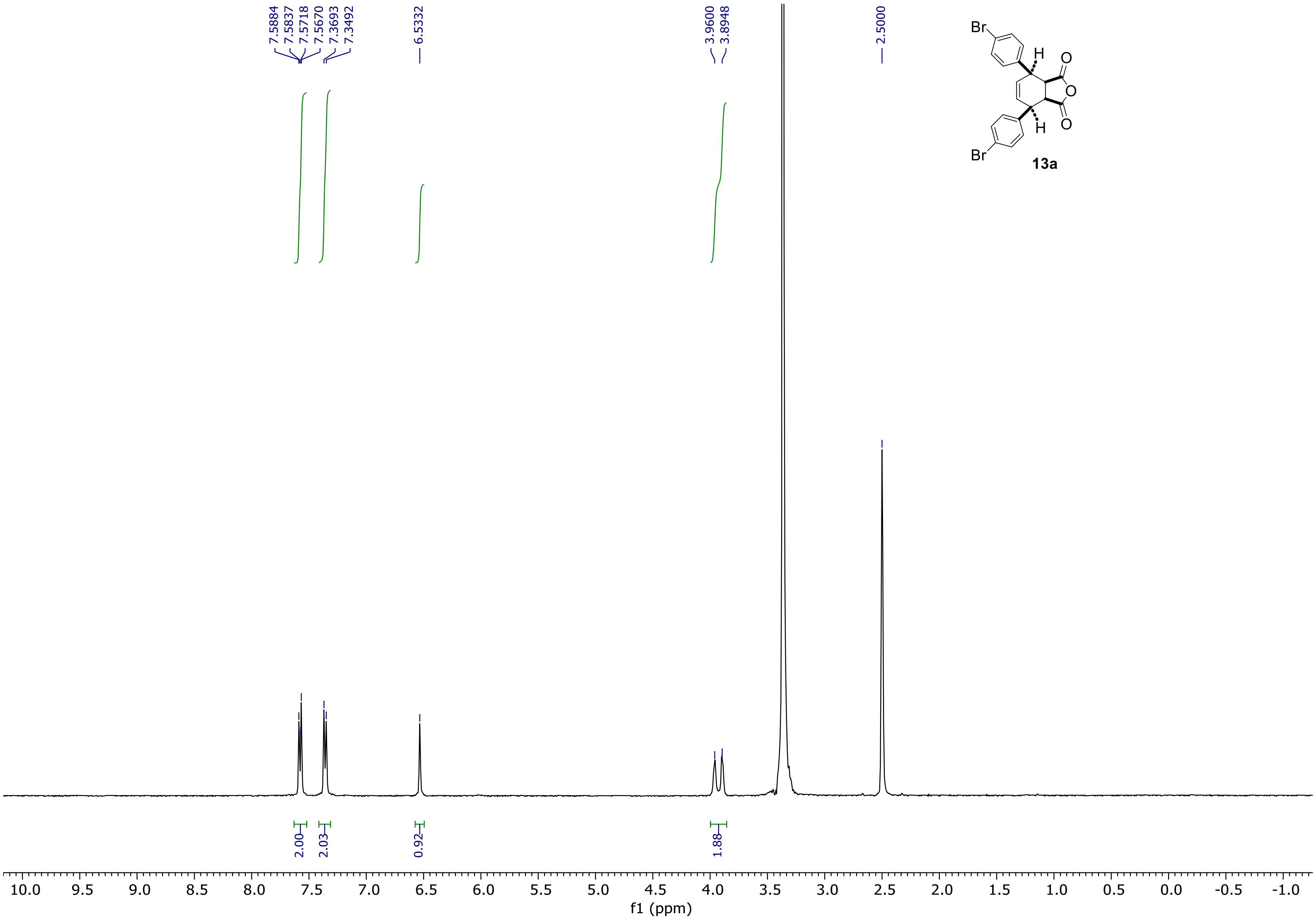
7.5884
7.5837
7.5718
7.5670
7.3693
7.3492
6.5332
3.9600
3.8948
2.5000
Br
H
O
O
O
H
Br
13a
2.00
2.03
0.92
1.88
10.0 9.5 9.0 8.5 8.0 7.5 7.0 6.5 6.0 5.5 5.0 4.5 4.0 3.5 3.0 2.5 2.0 1.5 1.0 0.5 0.0 -0.5 -1.0
f1 (ppm)

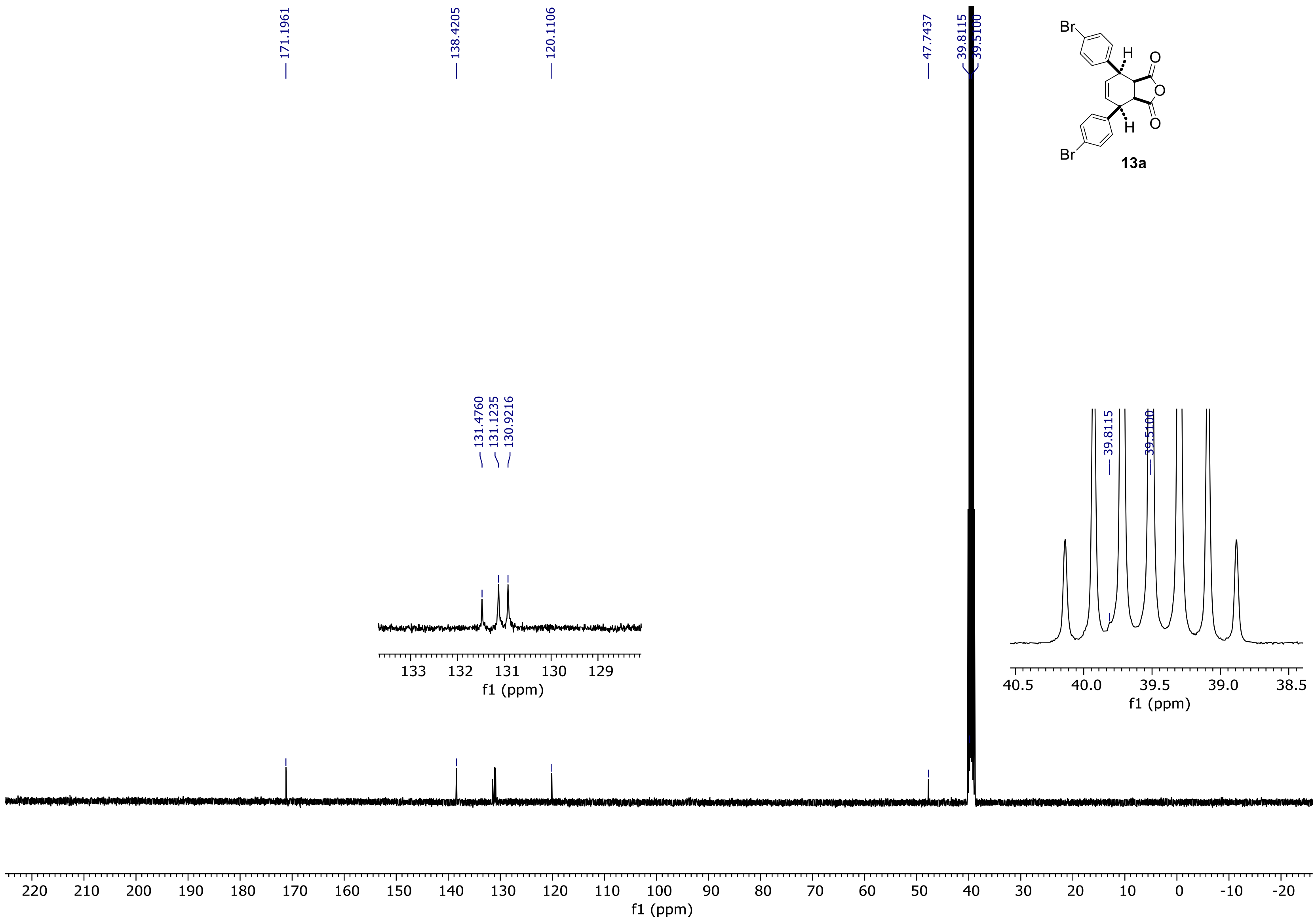
171.1961
138.4205
120.1106
47.7437
39.8115
39.5100
Br
H
O
O
O
H
Br
13a
131.4760
131.1235
130.9216
133 132 131 130 129
f1 (ppm)
39.8115
39.5100
40.5 40.0 39.5 39.0 38.5
f1 (ppm)
220 210 200 190 180 170 160 150 140 130 120 110 100 90 80 70 60 50 40 30 20 10 0 -10 -20
f1 (ppm)

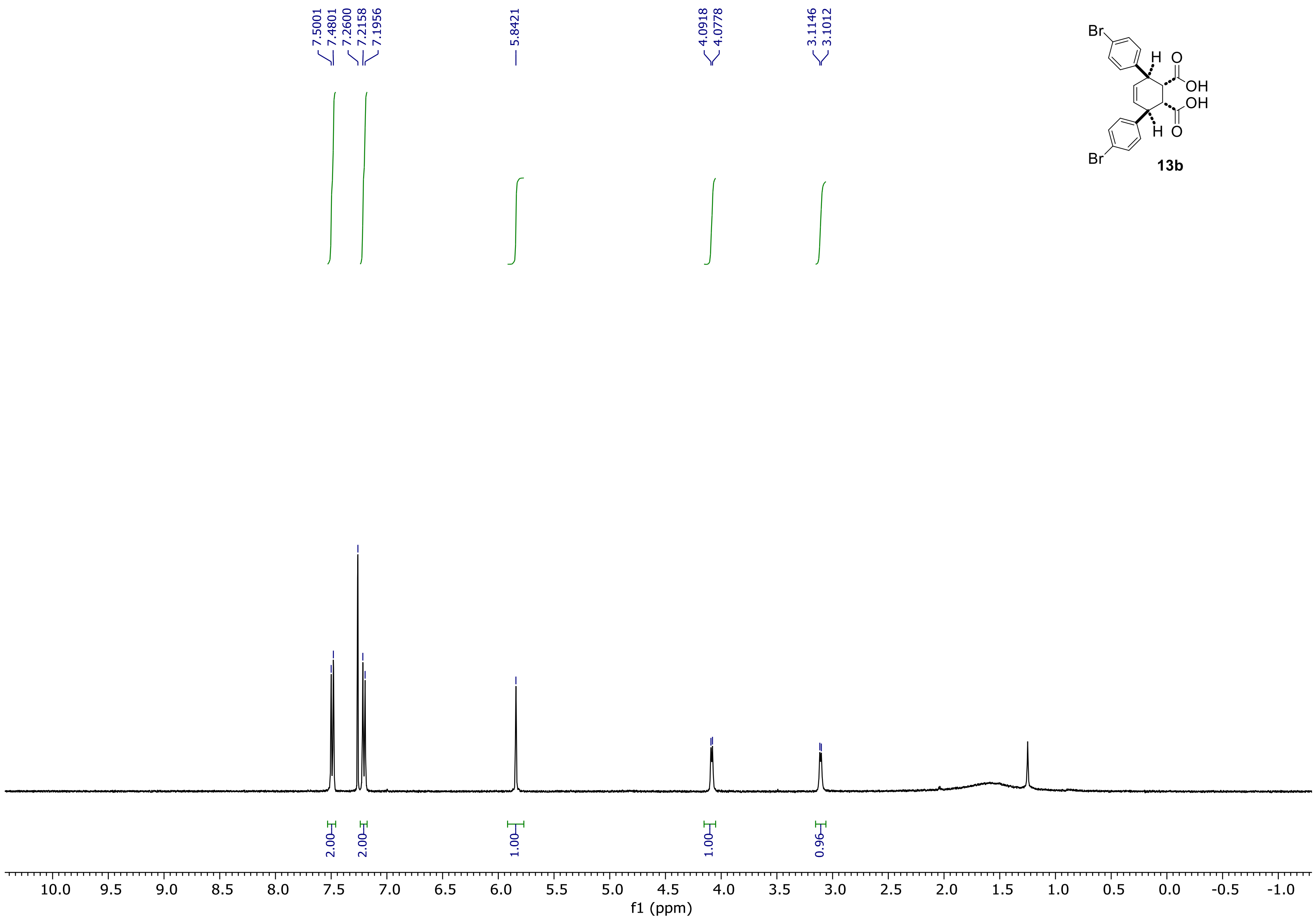
7.5001
7.4801
7.2600
7.2158
7.1956
5.8421
4.0918
4.0778
3.1146
3.1012
Br
H
O
OH
OH
H
O
Br
13b
2.00
2.00
1.00
1.00
0.96
10.0
9.5
9.0
8.5
8.0
7.5
7.0
6.5
6.0
5.5
5.0
4.5
4.0
3.5
3.0
2.5
2.0
1.5
1.0
0.5
0.0
-0.5
-1.0
f1 (ppm)

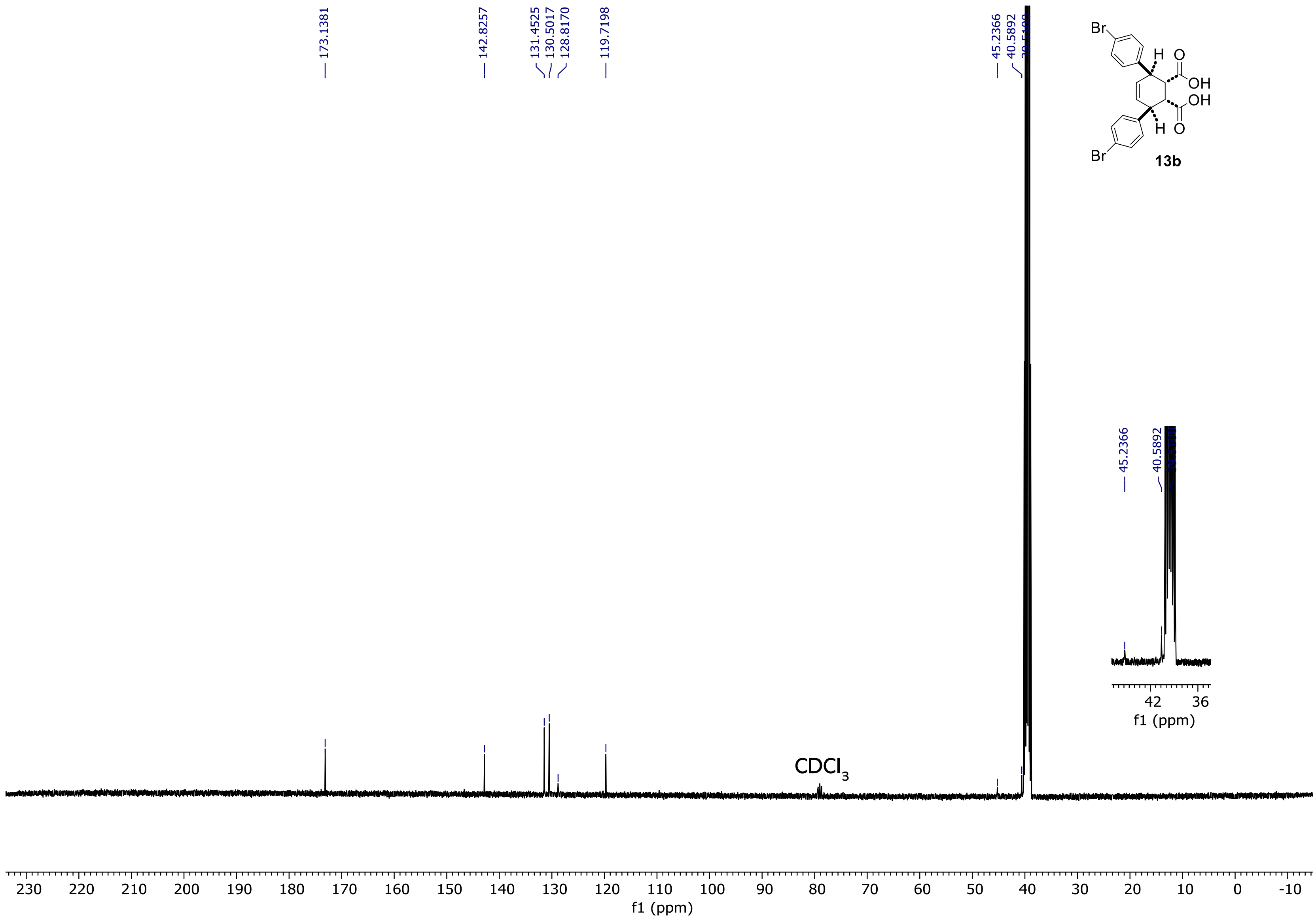
173.1381
142.8257
131.4525
130.5017
128.8170
119.7198
45.2366
40.5892
Br
H
O
OH
OH
H
O
Br
13b
45.2366
40.5892
42
36
f1 (ppm)
CDCl3
230
220
210
200
190
180
170
160
150
140
130
120
110
100
90
80
70
60
50
40
30
20
10
0
-10
f1 (ppm)

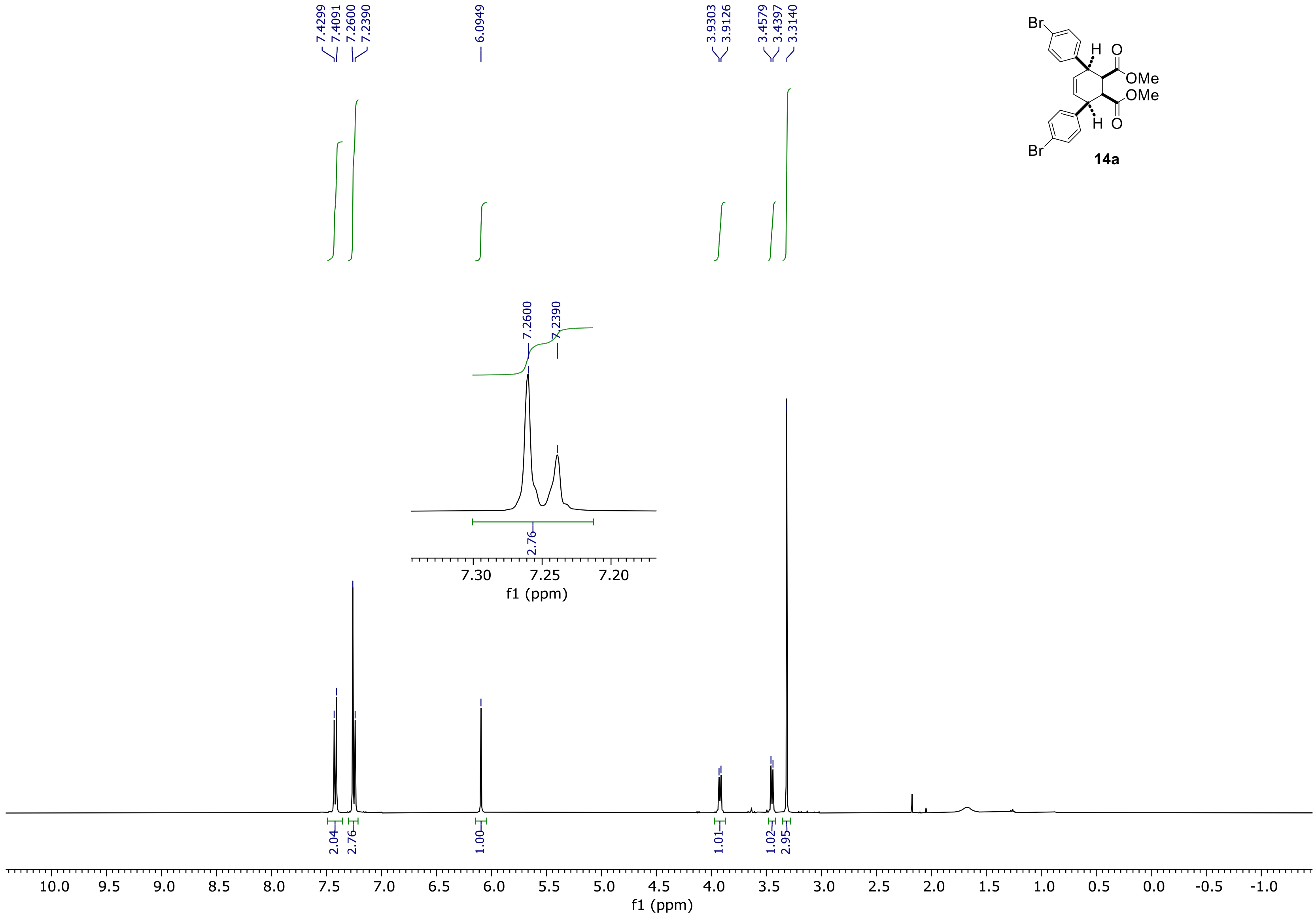
7.4299
7.4091
7.2600
7.2390
6.0949
3.9303
3.9126
3.4579
3.4397
3.3140
2.04
2.76
1.00
1.01
1.02
2.95
7.30 7.25 7.20
f1 (ppm)
10.0 9.5 9.0 8.5 8.0 7.5 7.0 6.5 6.0 5.5 5.0 4.5 4.0 3.5 3.0 2.5 2.0 1.5 1.0 0.5 0.0 -0.5 -1.0
f1 (ppm)
Br
H
O
OMe
OMe
H
O
Br

14a

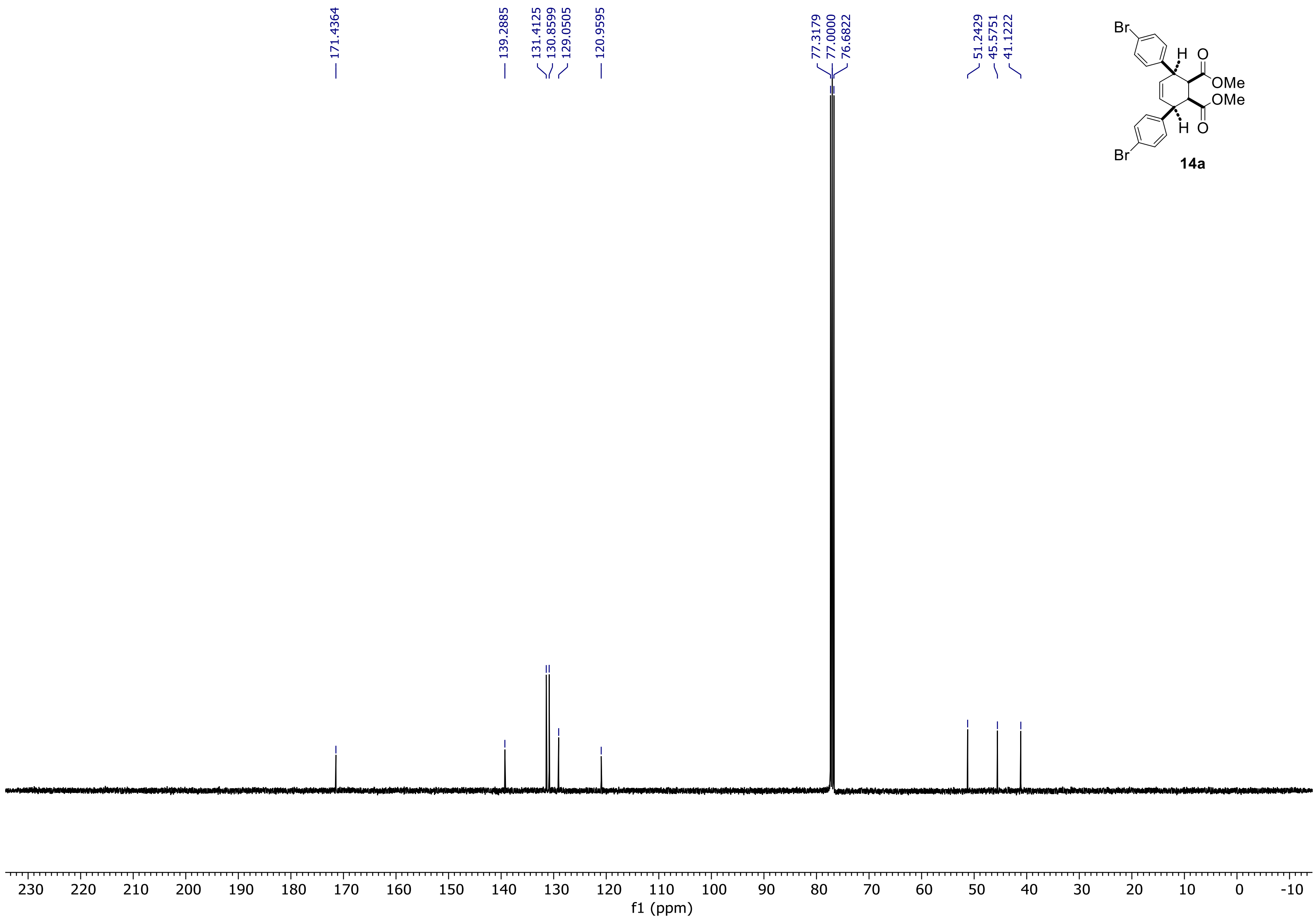
171.4364
139.2885
131.4125
130.8599
129.0505
120.9595
77.3179
77.0000
76.6822
51.2429
45.5751
41.1222
Br
H
O
OMe
OMe
H
O
Br
230 220 210 200 190 180 170 160 150 140 130 120 110 100 90 80 70 60 50 40 30 20 10 0 -10
f1 (ppm)
14a

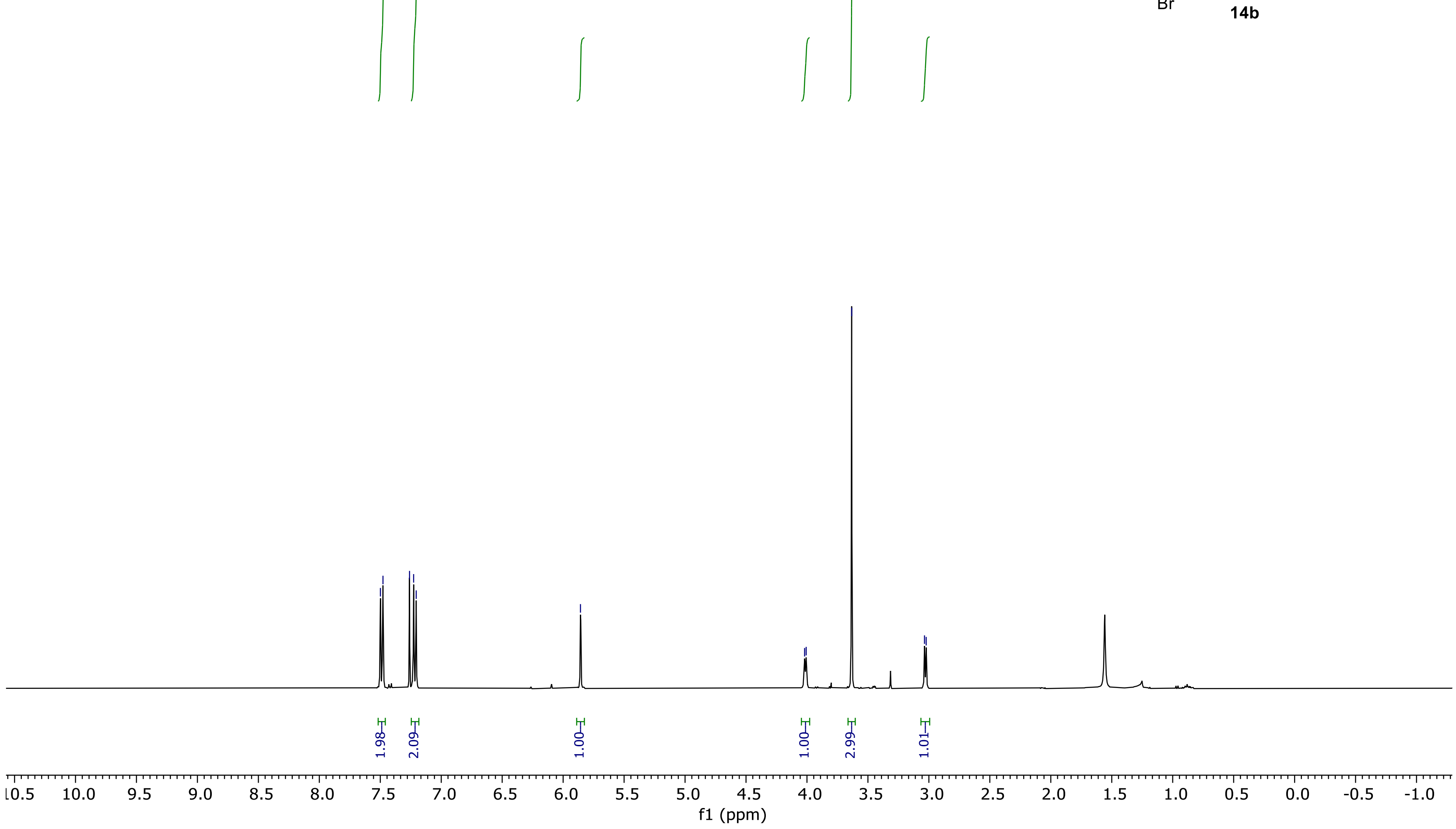
7.4990
7.4779
7.2600
7.2264
7.2053
5.8580
4.0194
4.0065
3.6330
3.0359
3.0222
1.98
2.09
1.00
1.00
2.99
1.01
10.5 10.0 9.5 9.0 8.5 8.0 7.5 7.0 6.5 6.0 5.5 5.0 4.5 4.0 3.5 3.0 2.5 2.0 1.5 1.0 0.5 0.0 -0.5 -1.0
f1 (ppm)

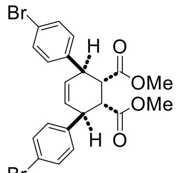
Br
H
O
OMe
OMe
H
O
Br

14b

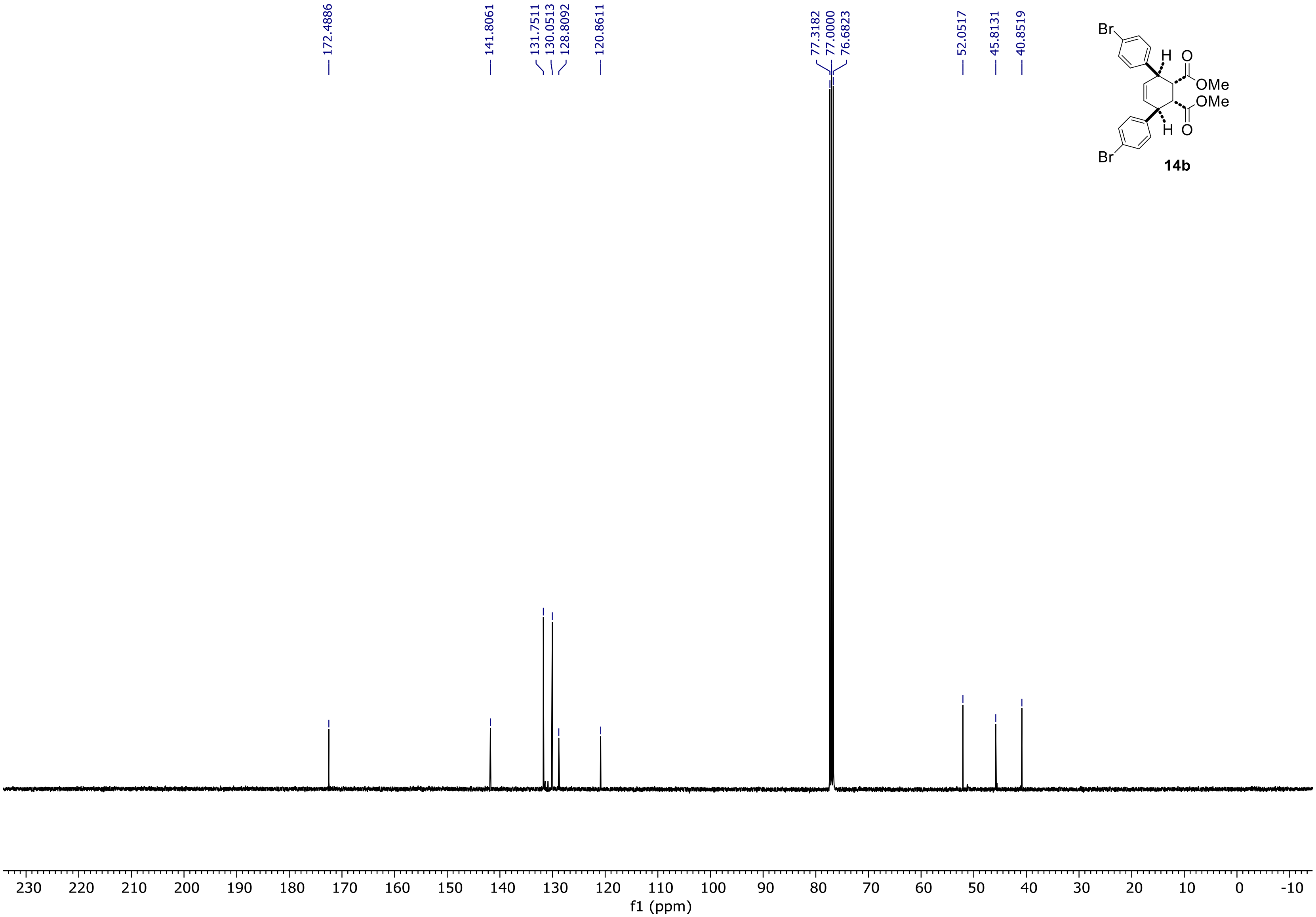
172.4886
141.8061
131.7511
130.0513
128.8092
120.8611
77.3182
77.0000
76.6823
52.0517
45.8131
40.8519
230 220 210 200 190 180 170 160 150 140 130 120 110 100 90 80 70 60 50 40 30 20 10 0 -10
f1 (ppm)
Br
H
O
OMe
OMe
H
O
Br

14b

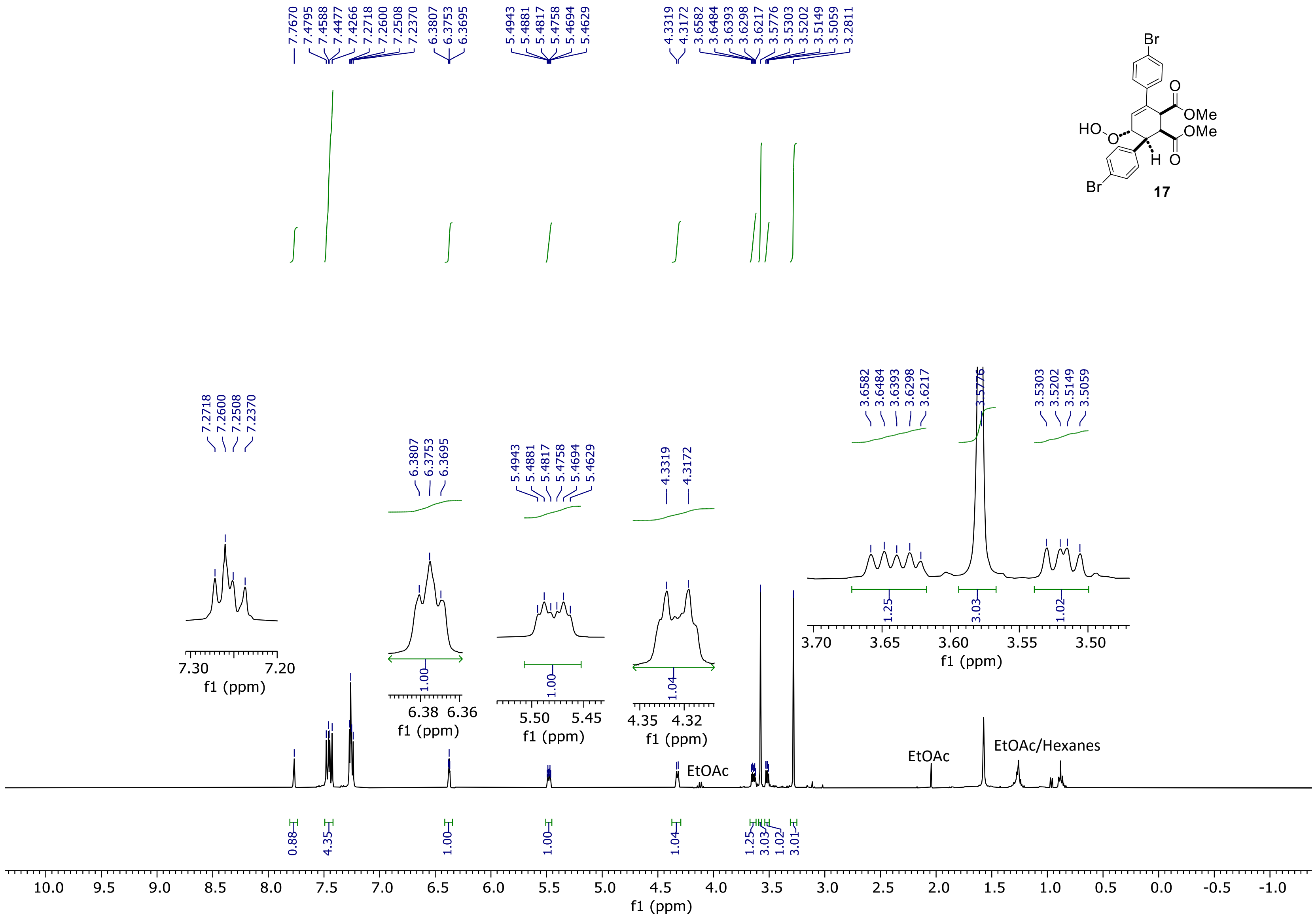

Br
HO
O
OMe
OMe
H
O
O
Br
17
EtOAc
EtOAc
EtOAc/Hexanes
f1 (ppm)

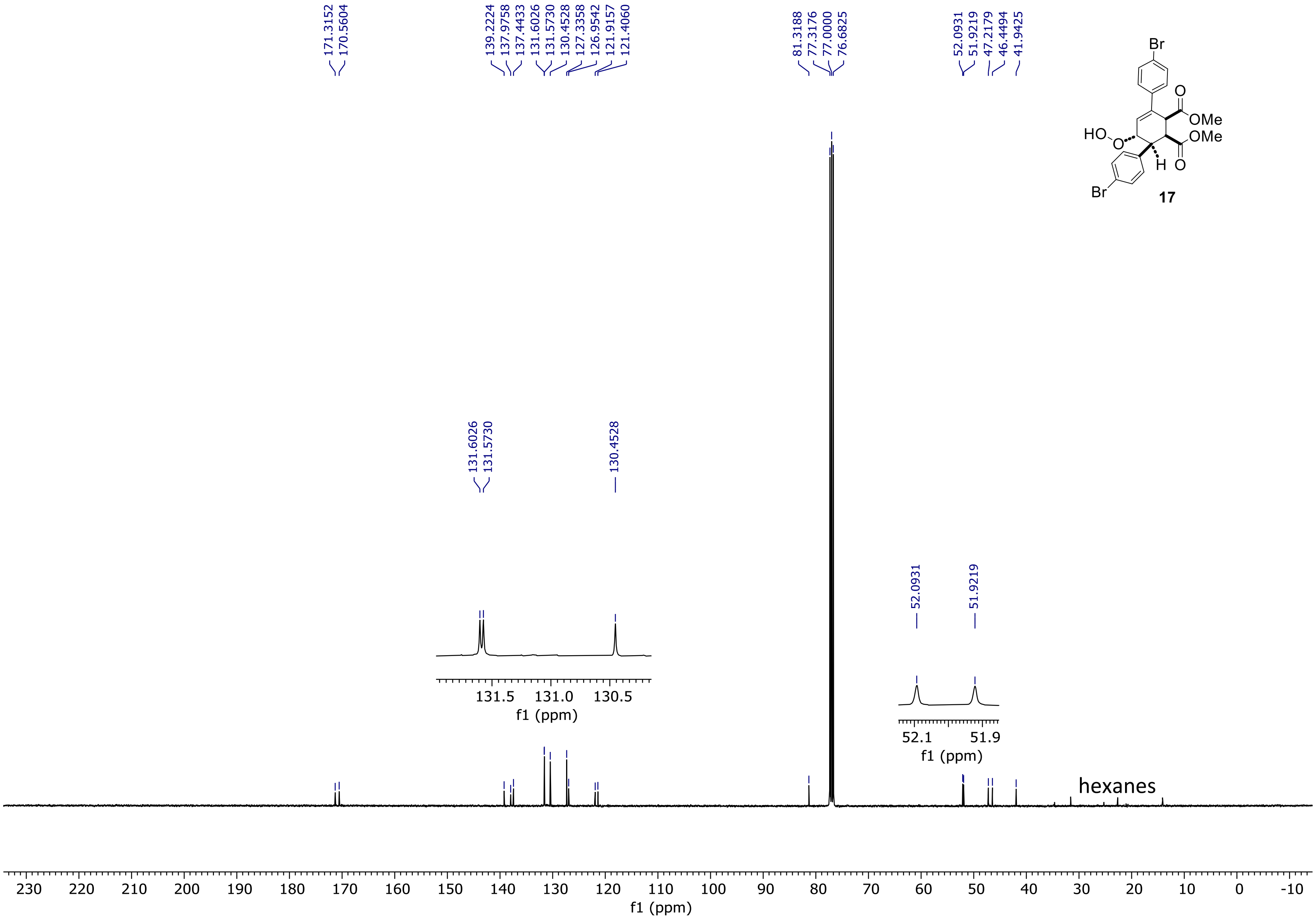
171.3152
170.5604
139.2224
137.9758
137.4433
131.6026
131.5730
130.4528
127.3358
126.9542
121.9157
121.4060
81.3188
77.3176
77.0000
76.6825
52.0931
51.9219
47.2179
46.4494
41.9425
Br
O
OMe
HO
O
OMe
H
O
Br
17
131.6026
131.5730
130.4528
131.5
131.0
130.5
f1 (ppm)
52.0931
51.9219
52.1
51.9
f1 (ppm)
hexanes
230 220 210 200 190 180 170 160 150 140 130 120 110 100 90 80 70 60 50 40 30 20 10 0 -10
f1 (ppm)
